# The influence of depth and a subsea pipeline on fish assemblages and commercially fished species

**DOI:** 10.1371/journal.pone.0207703

**Published:** 2018-11-26

**Authors:** Todd Bond, Julian C. Partridge, Michael D. Taylor, Tim F. Cooper, Dianne L. McLean

**Affiliations:** 1 The UWA Oceans Institute and School of Biological Sciences, The University of Western Australia, Crawley, Western Australia, Australia; 2 BHP, Perth, Western Australia, Australia; 3 The UWA Oceans Institute, The University of Western Australia, Crawley, Western Australia, Australia; 4 Oceans Graduate School, The University of Western Australia, Crawley, Western Australia, Australia; University of Sydney, AUSTRALIA

## Abstract

Knowledge of marine ecosystems that grow and reside on and around subsea oil and gas infrastructure is required to understand impacts of this offshore industry on the marine environment and inform decommissioning decisions. This study used baited remote underwater stereo-video systems (stereo-BRUVs) to compare species richness, fish abundance and size along 42.3 km of subsea pipeline and in adjacent areas of varying habitats. The pipeline is laid in an onshore-offshore direction enabling surveys to encompass a range of depths from 9 m nearshore out to 140 m depth offshore. Surveys off the pipeline were performed across this depth range and in an array of natural habitats (sand, macroalgae, coral reef) between 1 km and 40 km distance from the pipeline. A total of 14,953 fish were observed comprising 240 species (131 on the pipeline and 225 off-pipeline) and 59 families (39 on the pipeline and 56 off-pipeline) and the length of 8,610 fish were measured. The fish assemblage on and off the pipeline was similar in depths of <80 m. In depths beyond 80 m, the predominant habitat off-pipeline was sand and differences between fish assemblages on and off-pipeline were more pronounced. The pipeline was characterised by higher biomass and abundances of larger-bodied, commercially important species such as: *Pristipomoides multidens* (goldband snapper), *Lutjanus malabaricus* (saddletail snapper) and *Lutjanus russellii* (Moses’ snapper) among others, and possessed a catch value 2–3 times higher per stereo-BRUV deployment than that of fish observed off-pipeline. Adjacent natural seabed habitats possessed higher abundances of *Atule mate* (yellowtail scad), *Nemipterus* spp. (threadfin bream) and *Terapon jarbua* (crescent grunter), species of no or low commercial value. This is the first published study to use stereo-BRUVs to report on the importance of subsea infrastructure to commercially important fishes over a depth gradient and increases our knowledge of the fish assemblage associated with subsea infrastructure off north-west Australia. These results provide a greater understanding of ecological and fisheries implications of decommissioning subsea infrastructure on the north-west shelf, and will help better inform decision-making on the fate of infrastructure at different depths.

## Introduction

The global oil and gas (O&G) industry have been operating in offshore marine environments for over a century [1]. Hundreds of thousands of kilometres of pipeline have been laid and >7500 offshore structures including more than 900 large-scale O&G platforms are installed globally for the hydrocarbon industry [1–3]. This offshore infrastructure interacts with marine ecosystems and has been shown to have both positive and negative effects. Negative interactions with surrounding marine ecosystems can include: materials degradation, release of contaminants, facilitation of the introduction/spread of invasive marine species, and shipping hazards [4–6]. Benefits of offshore infrastructure include: provision of hard surfaces supporting colonisation and establishment of communities, increased diversity and abundance of marine fauna including top-predators and consequent benefit to fisheries [4, 7–10]. Advantages of retaining decommissioned marine infrastructure can be seen in ‘rigs to reefs’ programs in which decommissioned platforms are retained because of recognised benefits such as benthic habitat conservation, enhanced fisheries resources, and cost savings for the O&G industry [4, 11].

Across the globe, very little is known about interactions of fish with subsea pipelines, despite the prevalence of pipelines in our oceans and the importance of fish as indicators of environmental state and stability, as well as a food resource for humans [12, 13]. An exception is the work by [14] using submersible transect surveys in 95–235 m of water in the Santa Barbara Channel, Southern California, where they showed a 6 to 7 times higher abundance of some fish species living in close proximity to pipelines compared with fish living over the adjacent seafloor. On the north-west shelf of Australia, [15] conducted a comprehensive assessment of fish and habitats from footage collected by Remotely Operated Vehicles (ROV) on two subsea pipelines. With 5962 individual fish comprising 92 species observed on ROV footage, [15] suggested that pipelines on the north-west shelf likely offer significant habitat that supports a diversity and abundance of commercially targeted fish species. However, to understand properly whether pipelines can become habitats for fish, particularly in light of historical loss of natural habitat, research is required that compares assemblages on pipelines with those in adjacent natural habitats.

The deeper regions of this study area have experienced significant historical trawling by foreign vessels from 1959–1990 [16], with catches exceeding 37,000 tonnes and over 30,000 trawl hours being recorded at the peak of activities in 1973 [17]. Over the duration of this trawl fishery, catches of sponges and other macrobenthos declined simultaneously with a change in composition of the fish community [16]. Decreasing catches of high value snapper (Lutjanidae) and emperors (Lethrinidae), and increases in those of lizardfish (Synodontidae) and threadfin bream (Nemipteridae) were recorded. This shift was likely caused by pair-trawling which modified habitat, removing well-developed epibenthic habitat with which snappers and emperors are associated, resulting in a prevalence of sparser habitats with which synodontids and nemipterids are more typically associated [16]. There is now only a small active fishery on the north-west shelf of Australia, with combined line, trap and trawl fisheries accounting for just under 1,800 t in 2015, of which the trawl fishery accounts for the majority at 1,172 t, predominately acting in waters of 50–100 m depth [18]. Anecdotally, trap fisheries appear to benefit from pipelines in the region with reports of increased catches adjacent to pipelines, however current catch levels are well below (*ca*. 4%) historical levels and are considered to be a response to effort reductions [19]. In an environment that has previously been stripped of much of its complex benthic habitat structure in depths greater than 70 m, pipelines (in addition to platforms and wellheads) could have a role to play in supporting and maintaining the recovery of invertebrate habitats and previously exploited fish species.

In contrast to deeper historically trawled areas, inshore shallows of the Pilbara coast in north-west Australia possess a high diversity of fish and invertebrate species [19–22]. Seagrass, coral reefs, macroalgal, and mangrove habitats can be found along the Pilbara coast, and provide an array of habitats for fish [21]. Nearshore marine habitats, including macrophyte and sessile invertebrate assemblages, are likely to be vulnerable to coastal development pressures, with these habitats considered to be essential for recruitment of important fishery species [23]. Numbers of fish and fish species in this nearshore region are comparable to those observed in the lagoon and inter-reef waters of the Great Barrier Reef Marine Park [21]. Although strong associations between fish species and common habitats in north-west Australia have been documented [21], there is no published work that examines associations between fish and infrastructure in these nearshore regions.

A suitable method for comparing fish assemblages on and off pipelines across a large depth range is baited remote underwater stereo-video (stereo-BRUV). Stereo-BRUV can, given appropriate design, be deployed to full ocean depths and allow accurate measurements of fish length after camera calibration, providing important information on population size structure. The technique is economical, often using consumer-grade components housed in a polyvinyl chloride (PVC) housing within a steel frame, costing significantly less to build, maintain and use when compared to ROVs or submersibles. Further, many species will likely flee with the approach of an ROV or submersible, a behaviour that likely accounted for the absence of *Lethrinus punctulatus* (blue-lined emperor) and *Lutjanus erythropterus* (crimson snapper) in [15], despite these species being caught in fish traps in the region. Use of bait as an attractant in stereo-BRUV reduces the likelihood of these important commercial species being underrepresented. Stereo-BRUV is an alternative, non-destructive observational sampling technique that, unlike ROV’s, can quickly and relatively inexpensively sample large spatial areas and are well known to be an effective monitoring technique for targeted fish species including commercially important species, a particular focus of this research [24, 25].

This study used stereo-BRUVs to compare species diversity, abundance and sizes of fish on a 42.3 km long subsea pipeline with those observed in natural habitats of the Pilbara region in depths from 15–140 m. Particular focus was given to important commercial species. Relationships between fish species diversity, abundance and biomass, and a range of biological and physical variables (depth, distance to pipeline, dominant habitat) were examined to further understand the drivers determining the fish assemblage both on and off the pipeline. The ecological value of this pipeline to fish and fisheries in the region is discussed in addition to the far-reaching applicability of the study for providing knowledge on the potential environmental consequences of decommissioning this pipeline and those similar in the region.

## Materials and methods

### Ethics statement

All fish in the current study were recorded with video using non-destructive techniques. Bait was used to attract fish and comes with animal ethics approval from the University of Western Australia Animal Ethics Committee (RA/3/100/1420), chaired by Professor Hugh Barrett.

### Study site and survey equipment

**Fig 1:**
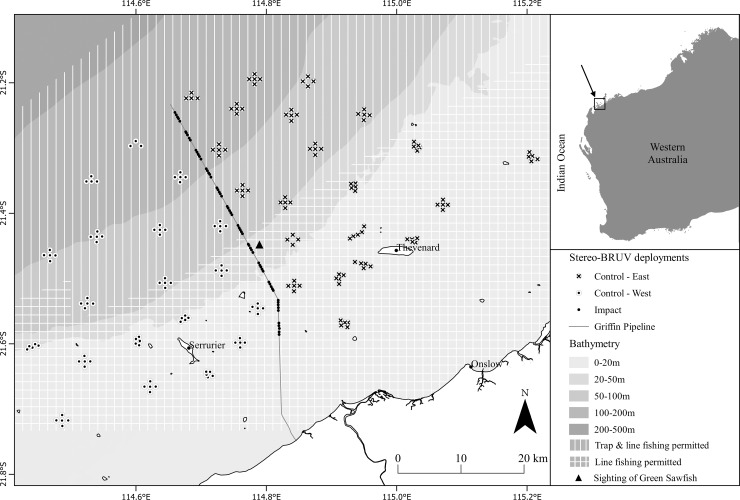
Location of all stereo-BRUV deployments and those analysed. Stereo-BRUV deployments, indicated in red, where visibility was less than 2 m, were excluded from analysis.

BHP’s Griffin pipeline, hereinafter referred to as the *pipeline*, is a 12 inch (*ca*. 300 mm) diameter pipeline that transported gas from the Griffin Field to the Australian mainland approximately 30 km south west of Onslow, a total distance of 62 km (Fig 1). Oil and gas production from the Griffin Field ceased in 2009 and the pipeline no longer transports gas to the shore. Surveys were performed from the pipeline’s inshore burial point (approximately 15 m water depth) and extended approximately 40 km either side of the pipeline offshore to 140 m water depth with survey locations bounding an area of approximately 2,500 km^2^ (Fig 1). Surveys were conducted over a ten-day period in March 2017 and a three-day period in April 2017 using baited remote underwater stereo-video systems (stereo-BRUVs). Stereo-BRUVs were deployed by the research vessel at sites on and off the pipeline in sets of five replicate deployments with neighbouring deployments separated by at least 400 m to reduce the likelihood of fish swimming between them within the one-hour sampling period (see [26]). Forty sites were sampled off-pipeline (19 control-west and 21 control-east) and twelve sites on-pipeline (Fig 1).

The pipeline was located from geographical information system shapefiles and coordinates provided by BHP and confirmed in the field using the vessel’s echo sounder. Although the pipeline is only 300 mm in diameter, it was easily seen on the sounder when not buried. If the pipeline was not visible on the sounder, it was assumed buried and the stereo-BRUV was deployed at the location provided. Each stereo-BRUV system comprised a pair of Canon Legria HGF25, high definition (1080i) video cameras set to record at 25 frames per second, or GoPro 3 Silver+, set to record at 1080p and 30 frames per second. For deployments in water deeper than 60 m, Canon Legria HGF25 cameras were used because of their performance advantages in low light conditions. Cameras were mounted on stereo-BRUV frames approximately 700 mm apart with their principal optical axes inwardly converged at 7° to maximise an overlapping field of view. Each camera was secured in a custom-built housing and mounted on a steel bar within a trapezium shaped frame that was tethered by rope to a surface buoy during deployments. This design maximises camera calibration, necessary for accurate size measurements, by minimising the movement of cameras during deployments and ensures alignment between deployments. Further information on the design and calibration of these systems can be found in [27, 28].

Four steel weight bars were added to each trapezium frame for deployments in depths >60 m to prevent systems overturning and a single blue LED-array light was added to provide illumination. The reaction of fish to different wavelengths of light is known to vary and various wavelengths have been used previously to visually sample fishes [29–32]. Here, blue light was chosen as it has been shown to provide a greater maximum illumination range than white light and red lights during tests in mid-western Australian waters [33]. An additional backwards facing GoPro and blue LED-array light was used to confirm the presence of the pipeline when behind the view of the main cameras and was not used to record fish abundance. Each stereo-BRUV was baited with approximately 1 kg of crushed pilchards (*Sardinops* spp.) contained within a plastic-coated wire mesh bait bag, positioned 1.2 m in front of the cameras on a metal bar. Each stereo-BRUV recorded for a minimum of 60 minutes on the seafloor. A fleet of 10 stereo-BRUVs was used to maximise field sampling efficiency.

### Stereo-video analysis

Stereo-BRUVs were calibrated using the CAL software package [34] and the analysis of video footage was facilitated through EventMeasure Stereo software [35]. Individual fish were identified to the lowest taxonomic level achievable. Some individuals could not be confidently or consistently identified to species level and were only identified to genus or family as a result. These genera included *Plectropomus* (coral trout), *Nemipterus* (threadfin bream), *Sillago* (whiting), *Scomberomorus* (mackerels) and *Decapterus* (scad), while the families included Apogonidae (cardinalfish), Bothidae (lefteye flounder), some Carangidae (trevally), Gerreidae (silverbiddy), Tripterygiidae (triplefin blenny) and Clupeidae (sprats). Relative abundance counts were obtained for each species (or lowest taxonomic level assigned). This was achieved by recording the maximum numbers of individuals, of each species, present within the field of view of the cameras at one time (MaxN) [36, 37]. The stereo-configuration of the video systems allows for accurate and precise lengths and distances to be measured [38]. Only fish within 7 m of the stereo-BRUV were counted in the MaxN and their length measured. Fork length of each fish contributing to a species’ MaxN was measured to the nearest mm while the fish was straight and at an angle between 45–90° to the cameras’ principal optical axes.

Fish with commercial value were a key focus for this study and included those most prevalent in trap, trawl and line fisheries operating in the Pilbara region of the North Coast Bioregion [18] (Table 1). These species are also typically targeted by recreational fishers in this region. The mass, in kg, of commercially targeted fish was calculated using the equation *a*L^*b*^ where *a* (g/cm^b^) is a parameter describing body shape and condition, L is length, and *b* indicates allometeric growth in body proportions. Values for *a* and *b* were obtained from FishBase [39] for each species where possible (Table 1). The commercial value of each species was calculated using the average beach price per kilogram [18] multiplied by biomass and summed across species to give a total value for each stereo-BRUV deployment.

**Table 1:** Commercial fishery species most prevalent in trap, trawl and line fisheries operating in the Pilbara region of the North Coast Bioregion.

Dependent variable	Common name	Beach price ($/kg)	*a*	*b*
Argyrops *spinifer*	frypan snapper	4.80	1.12E-04	2.65
*Epinephelus multinotatus*	rankin cod	8.43	1.67E-05	2.96
*Gymnocranius grandoculis*	Robinson's seabream	4.39	3.36E-05	2.87
*Lethrinus laticaudis*	grass emperor	7.33	4.01E-05	2.82
*Lethrinus nebulosus*	spangled emperor	5.41	1.87E-05	3.00
*Lethrinus olivaceus*	longnose emperor	5.93	2.94E-05	2.85
*Lethrinus punctulatus*	bluespotted emperor	4.37	2.94E-05	2.85
*Lutjanus erythropterus*	crimson snapper	5.18	2.44E-05	2.87
*Lutjanus malabaricus*	saddletail snapper	5.36	2.07E-05	2.92
*Lutjanus russellii*	Moses snapper	5.07	1.66E-05	2.98
*Lutjanus sebae*	red emperor	11.62	9.20E-06	3.21
*Lutjanus vitta*	brownstripe snapper	3.61	1.69E-05	2.98
*Plectropomus* spp.	coral trout	15.09	1.18E-05	3.06
*Pristipomoides multidens*	goldband snapper	8.81	2.00E-05	2.94

Beach prices were obtained from [18]. All a and b values are species-specific and taken from FishBase (Froese & Pauley 2017), with the exception of L. laticaudis taken from the congeneric Wattsia mossambica (Mozambique large-eye bream), Lethrinus punctulatus calculated from the family average from FishBase, and Plectropomus spp. which used the values from Plectropomus leopardus.

Dominant habitat type and vertical relief of habitat recorded on each stereo-BRUV deployment was assessed from stereo-video imagery using the program TransectMeasure [40]. Imagery was split into a 5 x 4 grid and dominant habitat cover characterised by applying an adapted version of the Collaborative and Automated Tools for Analysis of Marine Imagery (CATAMI) classification scheme for marine biota and substrates [41]. Habitat was selected from broad habitat types: unconsolidated (sand/rubble), consolidated (rocky bottom), hard corals, black and octocorals, sponges, crinoids, hydroids, seagrasses, ascidians and macroalgae. A further habitat variable, benthic biota, was created by combining all structurally complex habitat. If the pipeline was visible, it was recorded as consolidated, unless it was otherwise dominated by cover in any of the aforementioned CATAMI habitat types. For each deployment, a list of all dominant habitat types was recorded, this is referred to as ‘percentage dominance (%)’ rather than percentage cover as it is, in practice, a measure of how often each habitat type was observed to be the most prevalent in each of the 20 grid cells. Grid cells placed over open water were categorised as having ‘no biota’ and were excluded from the overall percentage dominance and final analyses. Each cell containing biota was also assigned a visual relief score from 1 to 5, where 1 indicates no vertical relief, such as flat sand or pebbly patches, and 5 indicates high structural complexity, such as caves or vertical walls. An average and standard deviation of relief was calculated for each deployment.

### Data analysis

This study had an asymmetrical design, with only one ‘impact’ location (pipeline) and multiple ‘control’ locations, defined here as those occurring to the east and west of the pipeline (see Fig 1). A three factor design was used to compare fish assemblages: Factor 1: Impact versus Control (IvC, fixed, with two levels; I = impact and C = control), Factor 2: Location (Loc, random, nested in IvC with two levels in control and one level in impact), and Factor 3: Site (random, nested in Loc(IvC) with multiple levels). There were five replicate stereo-BRUV deployments per site, with the exception of two sites east and one west of the pipeline that had four deployments, and one site west of the pipeline that had three. Data were analysed using a permutational analysis of variance with 9999 permutations (PERMANOVA) [42] in the PRIMER-E statistical software package [43] using the PERMANOVA+ add on [44]. For univariate data, analyses were conducted using the Euclidean Distance dissimilarity measure on untransformed species richness data and fourth-root transformed total abundance data (sum of all MaxN’s for each deployment). The same method was used for analysis of the total abundance, total biomass and total beach $ value of commercial species (each fourth-root transformed).

The multivariate relative abundance dataset and relative biomass datasets (each fourth-root transformed as the data set contained zero values for some species and numbers >100 for others) were analysed using Bray-Curtis dissimilarities. Principal coordinate ordination (PCO) [44], was used to construct an unconstrained ordination of both datasets, to investigate the significant term of IvC. Initial PCO analyses identified distinct separation of deployments by depth. To examine differences in fish assemblages on and off the pipeline, it is also important to consider depth effects. To do this, deployments were grouped into three depth categories; shallow <40 m, mid-depth 40–80 m, and deep >80 m. The shallow to mid-depth cut-off at 40 m was chosen because the inshore island archipelago stops at approximately 30 m and the north-west shelf begins. By chance, no stereo-BRUVs were deployed between 30–40 m, the deepest limit of shallow deployments being 29.6 m and the shallowest limit of mid-depth deployments being 40.1 m, and there is consequently a gap in our data in the 30–40 m depth band. The mid-depth to deep cut-off at 80 m was chosen as this was the approximate depth that natural daylight was invisible on stereo-BRUV footage and below this depth all video recordings relied on blue light illumination. A canonical analysis of principal coordinates (CAP) [45, 46] was undertaken using the new factor depth x IvC to constrain the data. Individual species that were likely responsible for any of the observed differences were identified using Spearman correlations of their relative abundance or relative biomass with the canonical axes. A correlation of |R| ≥ 0.5 was used as an arbitrary cut-off to display potential relationships between individual species and the axes.

For each depth zone, a PCO was constructed to examine the influence of IvC. Individual species that were likely responsible for any of the observed difference in each PCO were identified by examining Spearman correlations of their abundance with PCO axes. A correlation of |R| ≥ 0.5 was used as an arbitrary cut-off to display potential relationships between individual species and the axes. These species were identified as *key species* and univariate analyses were undertaken, as described below (generalised additive models), using their relative abundance to further understand their spatial distribution and interaction with the pipeline.

In depths <40 m, there was considerable variation in habitats off the pipeline. To examine how fish assemblages along the pipeline compared to those in differing habitats off the pipeline, we categorised each stereo-BRUV deployment by habitat as follows:

- *Off-pipeline sand*: off-pipeline deployments with ≥80% sand cover (77 deployments)
- *Off-pipeline benthic biota*: off-pipeline deployments with ≥20% benthic biota cover (22 deployments)
- *Pipeline sand*: pipeline deployments with ≥80% sand cover, where the pipeline could not be seen in the forward or rear facing cameras (seven deployments)
- *Pipeline benthic biota*: pipeline deployments where the pipeline was visible and/or ≥20% benthic biota cover (13 deployments).

A PCO was constructed and deployments identified by these habitat categories to visually interpret differences and similarities among various habitats on and off the pipeline in depths <40 m.

The minimum distance from the position of each stereo-BRUV deployment to the pipeline (herein after referred to as *minimum distance*) was calculated using the R package rgeos [47]. The influence of this distance and habitat variables (mean relief, standard deviation of relief, unconsolidated–sand/rubble, and benthic biota) on the relative abundance of key species identified in the CAP or PCO plots (MaxN), total abundance (sum all MaxN) and species richness (total number of species per deployment) was investigated for each depth category (<40 m, 40–80 m, >80 m) using generalised additive models (GAMs) [48]. A full subsets approach [49] was taken where all possible combinations of predictor variables were considered with a maximum number of predictor variables set to three. The complete set of possible models was reduced if estimated pairwise correlations between two predictors was > 0.28 [50].Model selection was based on Akaike Information Criterion (AIC) [51] and AIC weights (wAIC) [52]. Models that had AIC values which differed by less than two units show weak evidence for favouring one over the other [52, 53]. Therefore, the best model chosen was within two AIC of the lowest AIC value and had the lowest number of variables or was the most parsimonious [52]. For further detail on model selection and the full subsets approach used, see [49].

Kernel Density Estimates (KDEs) were created using the length-frequency data for all fish to investigate differences in the size structure of the fish assemblages on and off the pipeline within each depth category or separately for sand and benthic biota habitat types within the shallow depth category. Bandwidths were chosen via a “plug-in” style selection which does not make assumptions on the distribution of data [54] and were estimated using the ‘dpik’ function in the R package ‘KernSmooth’ [55]. Our statistical test compared the area between the two sets of KDEs using permutations of the data as random pairs and used the function ‘sm.density.compare’ in the R package ‘sm’ [56]. The null model is indicated on the plot as a grey band and represents one standard error above and below the null model. Lines outside of this band would indicate significant difference by location and the length likely causing any difference.

A heat map of predicted occurrence was generated using the model fits of spatial variables (latitude and longitude) for the biomass of commercial fish species. Spatial predicts were limited to within 10 km radius of deployments.

## Results

Three hundred and ten deployments were conducted between 9.3–135.3 m depth, however of these a total of 255 stereo-BRUV deployments had sufficient visibility (>2 m) for analysis, 60 on the pipeline and 195 off-pipeline.

### Fish assemblage description

**Fig 2:**
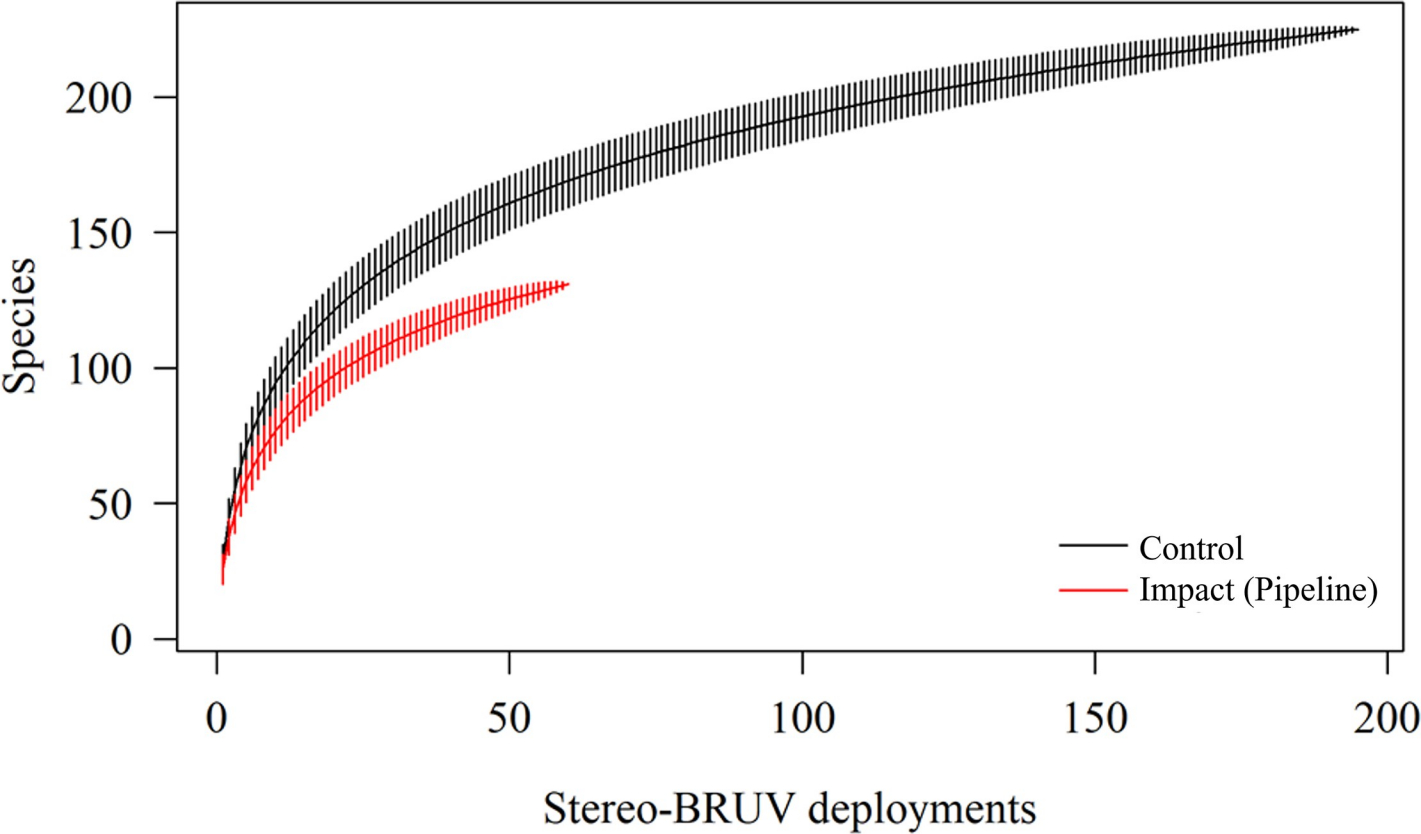
Species accumulation (rarefaction) curves for each location.

A total of 14,953 fish were observed from 240 species (131 on the pipeline and 225 off-pipeline) and 59 families (39 on the pipeline and 56 off-pipeline). Mean abundance, species richness and length measures are presented in Table 2. Fifteen species were observed only on the pipeline while 96 were unique to deployments off-pipeline. Comparisons of the total number of species should be made with caution given the disproportionate sampling effort on (n = 195 deployments) and off the pipeline (n = 60). Species accumulation (rarefaction) curves showed that for an equivalent sampling effort, species richness was higher at control locations (off-pipeline) (Fig 2). A complete list of all bony and cartilaginous fish species observed, their feeding guild, relative abundance and commonality is included in Supplementary Information (S1 and S2 Tables). The lengths of 8,610 fish were measured, the smallest a 21 mm juvenile *Lethrinus* spp. (unidentifiable juvenile emperor) and the largest was a 3.36 m *Galeocerdo cuvier* (tiger shark). A green sawfish (*Pristis zijsron)* was likely larger, but its length could only be estimated at ca. 3.76 m because it was greater than the field of view of the cameras and therefore measured in sections.

**Table 2:** The mean relative abundance (sum of MaxN), species richness (# species) and mean fish length (fork length; mm) of fish recorded on stereo-BRUV deployments on and off-pipeline.

Depth	Location	Number of deployments	Relative abundance (mean ± SE)	Species richness (mean ± SE)	Mean fish length (mm) (mean ± SE)
All	Pipeline	60	49.63 ± 7.02	10.76 ± 0.82	260.68 ± 4.57
	Off-pipeline	195	61.41 ± 5.93	10.87 ± 0.56	209.82 ± 2.02
<40 m	Pipeline	20	91.2 ± 15.78	15.85 ± 1.54	189.85 ± 4.04
	Off-pipeline	99	89.71 ± 8.77	14.90 ± 0.92	190.22 ± 2.07
40–80 m	Pipeline	13	48.15 ± 10.64	10.92 ± 1.50	313.74 ± 10.93
	Off-pipeline	35	63.17 ± 17.46	7.86 ± 0.51	276.47 ± 7.54
>80 m	Pipeline	27	19.56 ± 2.18	6.70 ± 0.44	415.70 ± 11.11
	Off-pipeline	61	14.48 ± 1.43	6.05 ± 0.20	312.38 ± 7.02

The ten most ubiquitous species on the pipeline were *Nemipterus* spp. (threadfin bream), *Pristipomoides multidens* (goldband snapper), *Argyrops spinifer* (frypan snapper), *Carangoides caeruleopinnatus* (onion trevally), *Lutjanus malabaricus* (saddletail snapper), *Choerodon cauteroma* (bluespotted tuckfish), *Pentapodus porosus* (northwest whiptail), *Lutjanus sebae* (red emperor), *Parupeneus heptacanthus* (cinnabar goatfish), *Abalistes stellatus* (starry triggerfish), and *Parupeneus indicus* (yellowspot goatfish) (S1 Table). The ten most ubiquitous species off the pipeline were *Nemipterus* spp., *P*. *porosus*, *Scomberomorus spp*., *C*. *caeruleopinnatus*, *Lagocephalus lunaris* (lunartail puffer), *Selaroides leptolepis* (yellowstripe scad), *Saurida undosquamis* (brushtooth lizardfish), *A*. *stellatus*, *Decapterus* sp1 (unidentified scad), and *A*. *spinifer* (S1 Table).

### Depth-partitioned fish assemblage

Statistical analysis of the multivariate relative abundance and biomass datasets (S3 Table) showed significant variability among sites (both p<0.01), but variation among control locations was not detected over and above this site-level variability (Loc(IvC) both p >0.5). The fish assemblage present along the pipeline (impact site) at the time of this study was distinct, in terms of relative abundance, from those observed at control locations (IvC: p *=* 0.014). However, fish assemblage biomass did not differ among locations or between the pipeline and control sites (p > 0.05).

**Fig 3:**
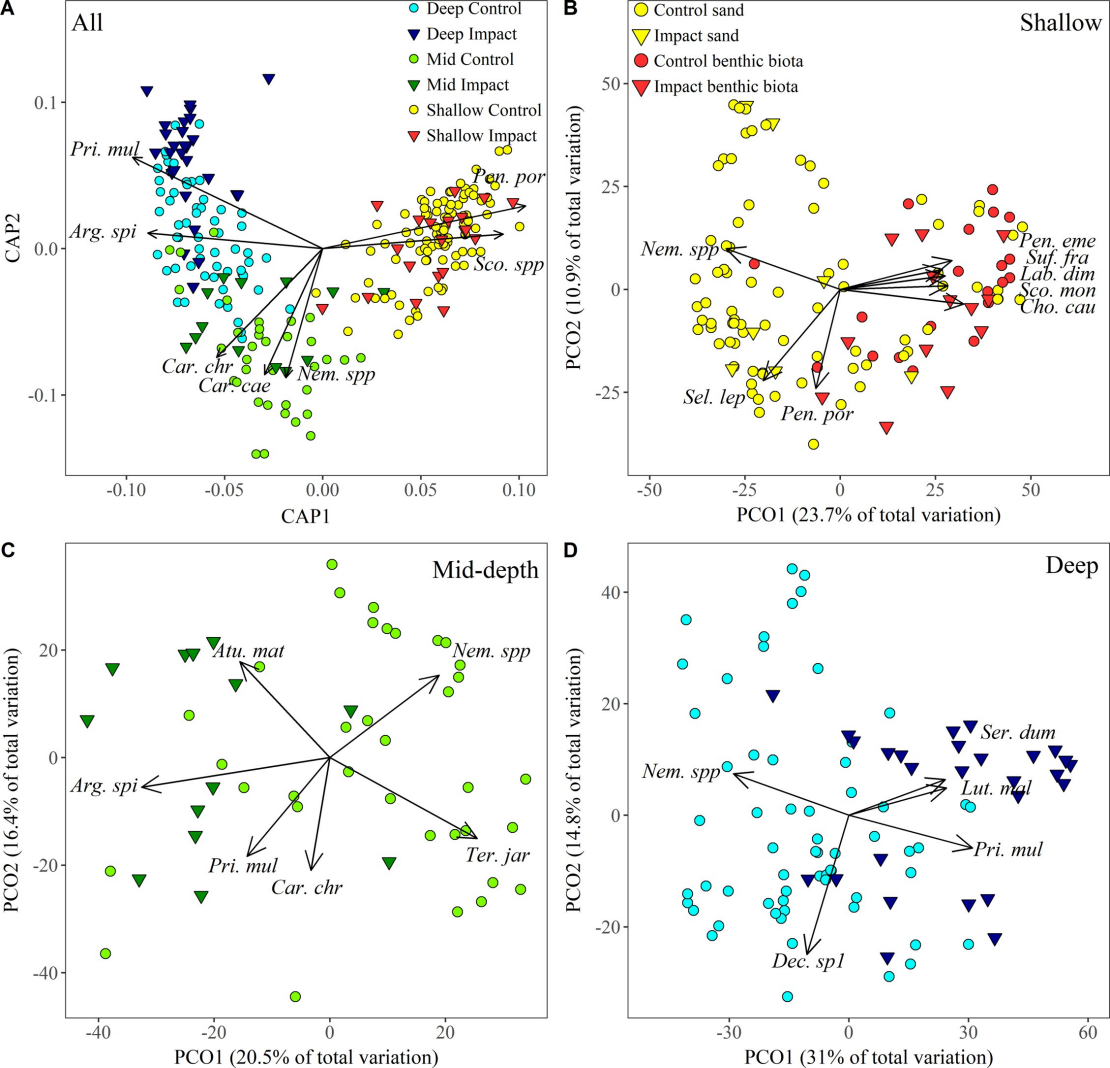
CAP and PCO plots using the relative abundance of fish. A) A CAP plot illustrating the grouping of deployments using the combined factor Location x Depth displays separation of depths; B-D) PCO plots illustrating patterns in the relative abundance of fish assemblages in depths < 40 m (B), in 40–80 m (C) and in >80 m (D). Species with a Spearmen correlations of |R| ≥ 0.5 (A), |R| ≥ 0.6 (B, D) and |R| ≥ 0.65 (C) to either axis are overlaid with vectors indicating the strength of the correlation (longer vectors indicate higher |R|) and the direction in which each species is shaping the distribution of samples.

A CAP analysis, constrained by the combined factor depth x IvC, shows separation across depths and species contributing to this separation (Fig 3A). These include *P*. *porosus* and *Scomberomorus* spp. in <40 m depth; *Carangoides chrysophrys* (longnose trevally), *Nempiterus* spp., and *C*. *caeruleopinnatus* in 40–80 m depth; and *A*. *spinifer* and *P*. *multidens* in >80 m depth. The degree of distinctiveness of each depth x IvC assemblage was illustrated by an overall CAP leave-one-out allocation success rate of 72.16%. Whole-of-study-area analyses demonstrating the overarching influence of depth (using GAMs) and the abundance distribution of key species and of species richness and total abundance is presented in Supplementary information (S4 Table; S1–S3 Figs).

Mean species richness and mean total relative abundance of fish declined with increasing depth, for both pipeline and off-pipeline deployments (Table 2). Mean species richness for depth-partitioned data was higher on the pipeline than off in all depth regions (Table 2). Mean relative abundance per deployment was higher on the pipeline in <40 m depth and in depths >80 m but lower than adjacent habitats in 40–80 m depth (Table 2). Fish were, on average, nearly twice as large on deployments in depths >80 m than in depths <40 m (Table 2).

### Fish-pipeline associations in depths <40 m

In depths <40 m, the five most ubiquitous species on the pipeline were *C*. *cauteroma* (70% deployments), *P*. *indicus* (60%), *S*. *monogramma* (55%), *S*. *leptolepis* (55%), and *A*. *stellatus* (50%). The five most ubiquitous species observed off-pipeline were *Scomberomorus* spp. (67%), *S*. *leptolepis* (51%), *Nemipterus* spp. (41%), *C*. *cauteroma* (34%), and *Parapercis nebulosa* (pinbanded grubfish; 30%).

**Fig 4:**
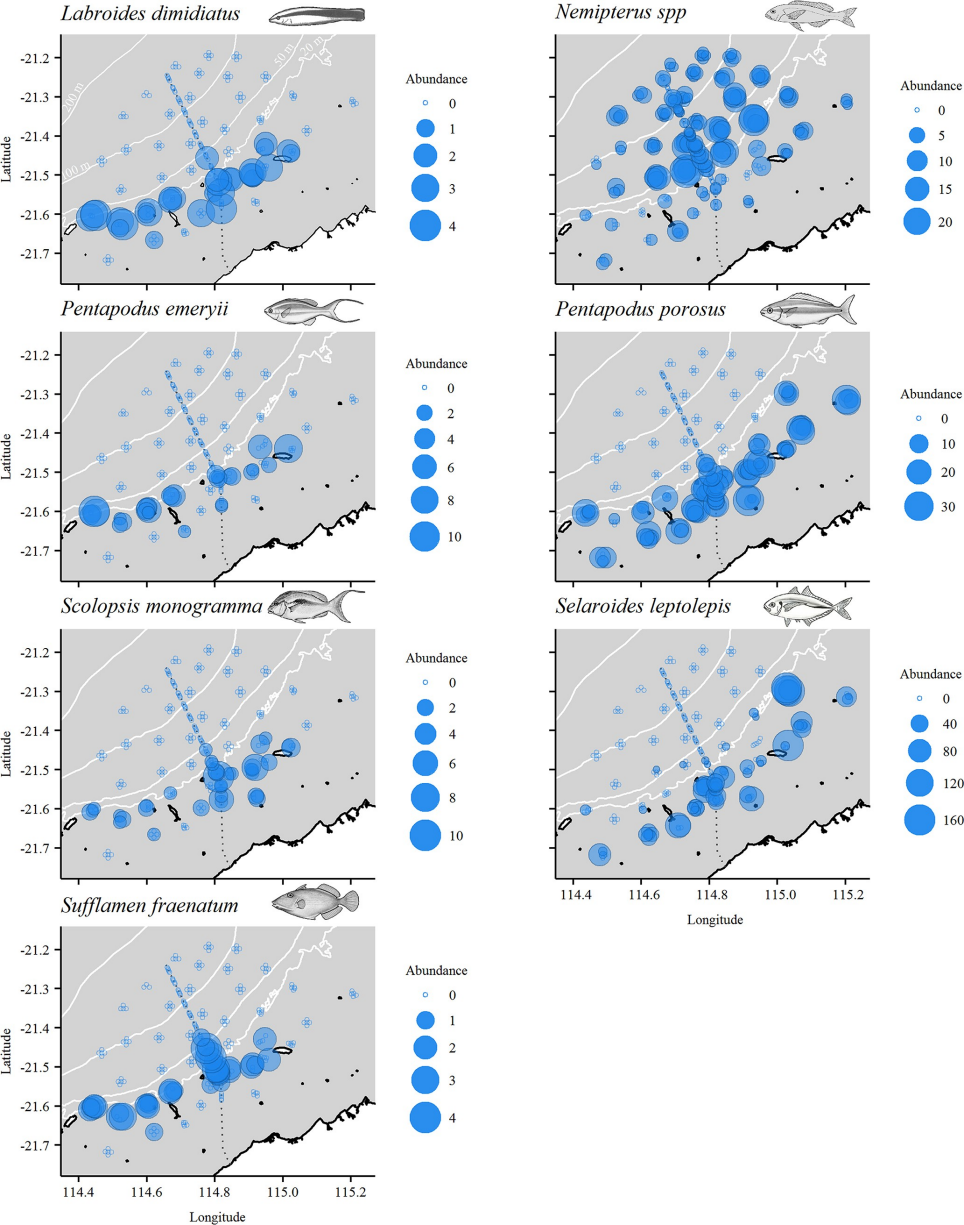
Spatial distribution of the relative abundance of key species on deployments in <40 m depth. MaxN bubble sizes reflect exact abundance, and therefore bubble size may be larger or smaller than those shown in the legend’s categories.

Multivariate analysis of the relative abundance of fish in the shallows (S3 Table) showed significant variability among sites (Site(Loc(IvC)): d.f. = 21, 118, MS = 6355.3, Pseudo-F = 2.67, p < 0.01), but no variation among control locations over and above this site variability (Loc(IvC): p = 0.14). There was also no difference in fish assemblages on and off the pipelines in depths <40 m (IvC: p = 0.45). The same result was obtained for biomass, with only significant variability among sites observed (p<0.001). A PCO plot, with deployments colour-coded by broad habitat categories observed in the shallows, shows separation of sand and benthic biota deployments, but no differences between pipeline and off-pipeline deployments in these habitats (Fig 3B). The separation between sand and benthic biota deployments (PCO1 axis) explains 23.7% of the total variation between all deployments. *Nemipterus* spp., *P*. *porosus* and *S*. *leptolepis* were correlated strongly with deployments in sand while *Pentapodus emeryii* (double whiptail), *Sufflamen fraenatum* (masked triggerfish), *Labroides dimidiatus* (common cleanerfish), *S*. *monogramma*, and *C*. *cauteroma* were correlated with benthic cover (Fig 3B). The spatial distribution of abundances of these key species in depths <40 m are presented in Fig 4 and further explored using GAMs.

**Fig 5:**
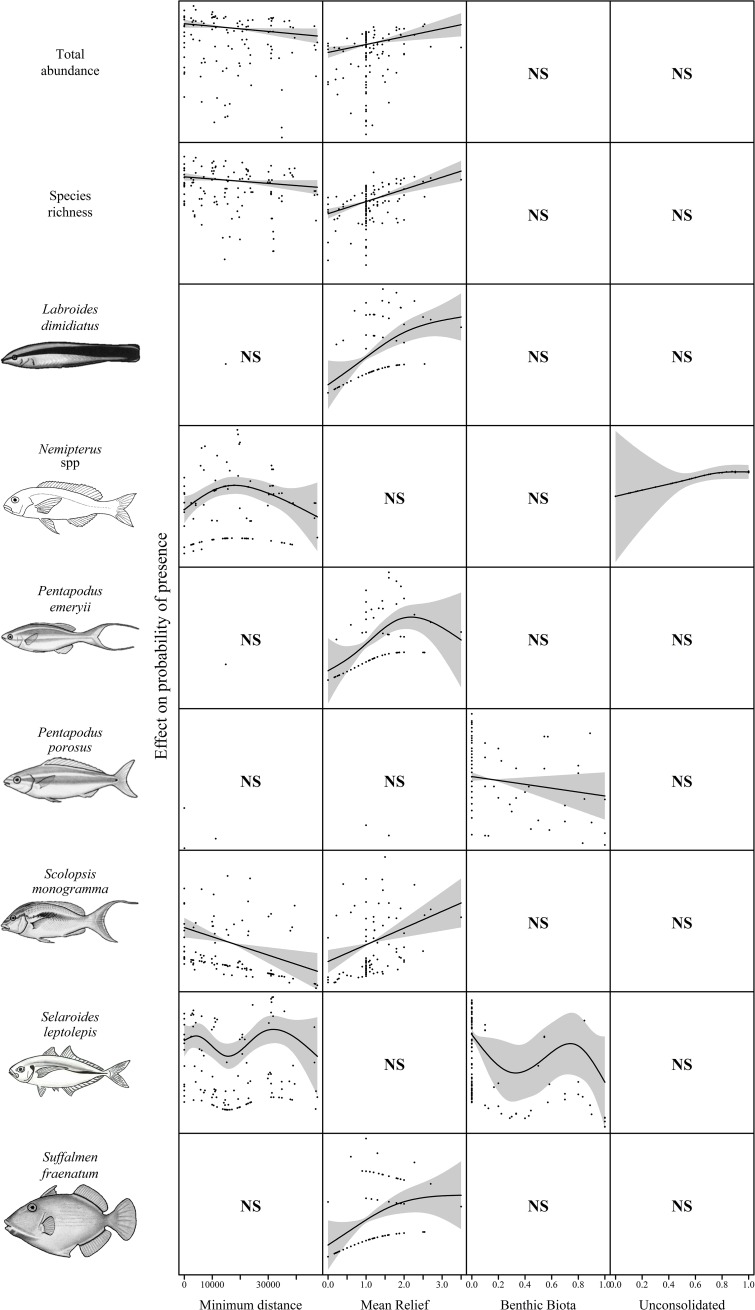
The abundance of key species in the shallow deployments relative to the most parsimonious explanatory variables (Table 3). The solid black line represents the estimated smoothing curve and dashed lines represents ±2 x SE of the estimate. Where a Null model was reported a second model was plotted if it was within 2 AIC on the Null model. ‘NS’ indicates the variable was not significant in the model.

GAMs were used to investigate relationships between species richness, total abundance and the abundance of key species shown in Fig 3B with the variables; minimum distance, mean relief, standard deviation of relief, unconsolidated (sand/rubble), and benthic biota (Table 3). Minimum distance was a predictor variable chosen in five of the best models. Total abundance, species richness, and the abundance of *S*. *monogramma* declined with increasing distance from the pipeline and *Nemipterus* spp. and *S*. *leptolepis* were low in abundance close to the pipeline and peaked in abundance at mid-distances (Figs 4 and 5). Total relative abundance and the relative abundance of *S*. *fraenatum*, *L*. *dimidiatus*, and *P*. *emeryii* increased with higher values of mean relief, an indicator of structural complexity (Fig 5). Conversely, *Nemipterus* spp. decreased in relative abundance as mean relief increased.

**Table 3:** Generalised additive models (GAMs) for predicting total abundance, species richness, and the abundance of key species in <40 m depth within 2 AIC of the top model.

	eDF	AIC	wAIC	R^2^	Best Model
Total Abundance	3.00	1296.99	0.67	0.10	Minimum distance + Mean relief
Species Richness	3.00	797.51	0.63	0.23	Minimum distance + Mean relief
*Choerodon cauteroma*	1.00	318.15	1.00	0.00	Null
*Labroides dimidiatus*	2.91	223.22	0.52	0.25	Mean relief
*Nemipterus* spp.	5.17	401.13	0.23	0.32	Minimum distance + Unconsolidated
*Pentapodus emeryii*	3.48	257.07	0.41	0.21	Mean relief
*Pentapodus porosus* (1)	1.00	737.77	0.19	0.00	Null
(2)	2.00	737.71	0.20	0.01	Benthic biota
*Scolopsis monogramma*	3.00	245.57	0.53	0.14	Minimum distance + Mean relief
*Selaroides leptolepis*	7.18	727.72	0.23	0.15	Minimum distance + Benthic biota
*Sufflamen fraenatum*	2.86	230.18	0.34	0.11	Mean relief

eDF, estimated degrees of freedom; AIC, Akaike Information Criterion; wAIC, Weighted Akaike Information Criterion. If the best model was Null, the second best model was reported if it was within 2 AIC of the Null model.

### Fish-pipeline associations in 40–80 m depth

In depths of 40–80 m, the five most ubiquitous species on the pipeline were *A*. *spinifer* (92% deployments), *Nemipterus* spp. (92%), *C*. *caeruleopinnatus* (62%), *P*. *multidens* (54%), and *Atule mate* (yellowtail scad, 46%). The five most ubiquitous species observed off-pipeline were *Nemipterus* spp. (94%), *C*. *caeruleopinnatus* (77%), *C*. *chrysophrys* (57%), *A*. *stellatus* (46%), and *A*. *spinifer* (34%) (S1 Table).

**Fig 6:**
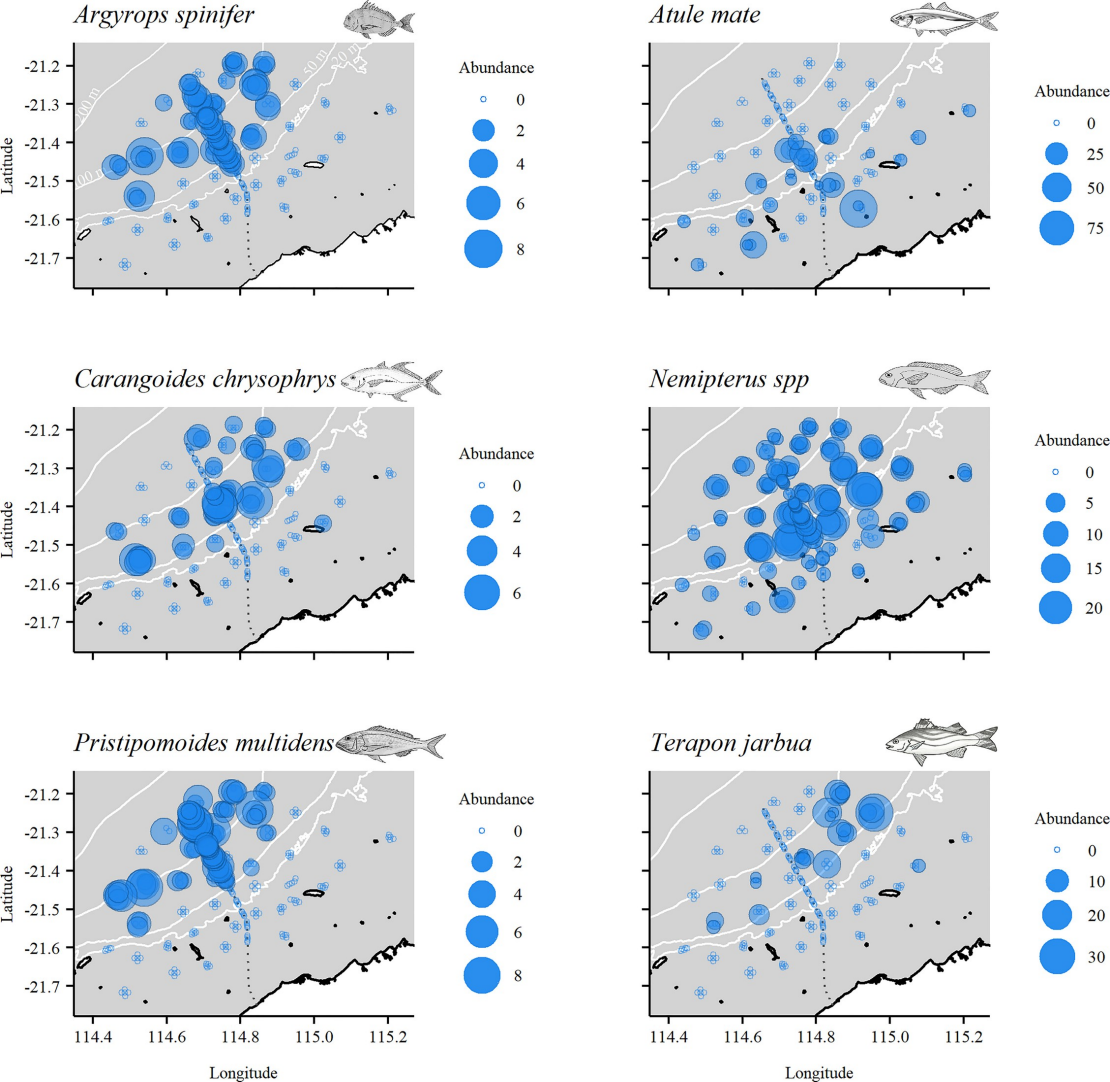
Spatial distribution of the relative abundance of key species on deployments in 40–80 m depth. MaxN bubble sizes reflect abundance, and therefore bubble size may be larger or smaller than those in the legend.

Multivariate analysis of fish relative abundance in 40–80 m depth (S3 Table) showed high variability among sites (Site(Loc(IvC)): d.f. = 7, 47, MS = 4657, Pseudo-F = 2.79, p < 0.01) but no difference among controls over and above this site variability Loc(IvC) (p = 0.62). In this mid-depth range fish assemblages were similar between impact (pipeline) and control (off-pipeline) locations (IvC: p = 0.07). The same result was obtained for the multivariate biomass data in this depth range (only significant variability among sites). Despite these non-significant results, Fig 3C shows some separation of pipeline and off-pipeline deployments, primarily in PCO1 axis which explained 20.5% of variation among deployments. Unlike deployments in <40 m depth, habitats off the pipeline in 40–80 m were predominantly sand (90.2%) and therefore not plotted in Fig 3C. The species that were responsible for separation between pipeline and off-pipeline deployments were *A*. *spinifer*, *C*. *chrysophrys*, and *P*. *multidens* associated with the pipeline and *A*. *mate*, *Nemipterus spp*. and *Terapon jarbua* (crescent grunter) with off-pipeline deployments (Fig 3C). *Terapon jarbua* was not recorded on the pipeline and was observed on 34% of deployments off-pipeline (Fig 6).

**Fig 7:**
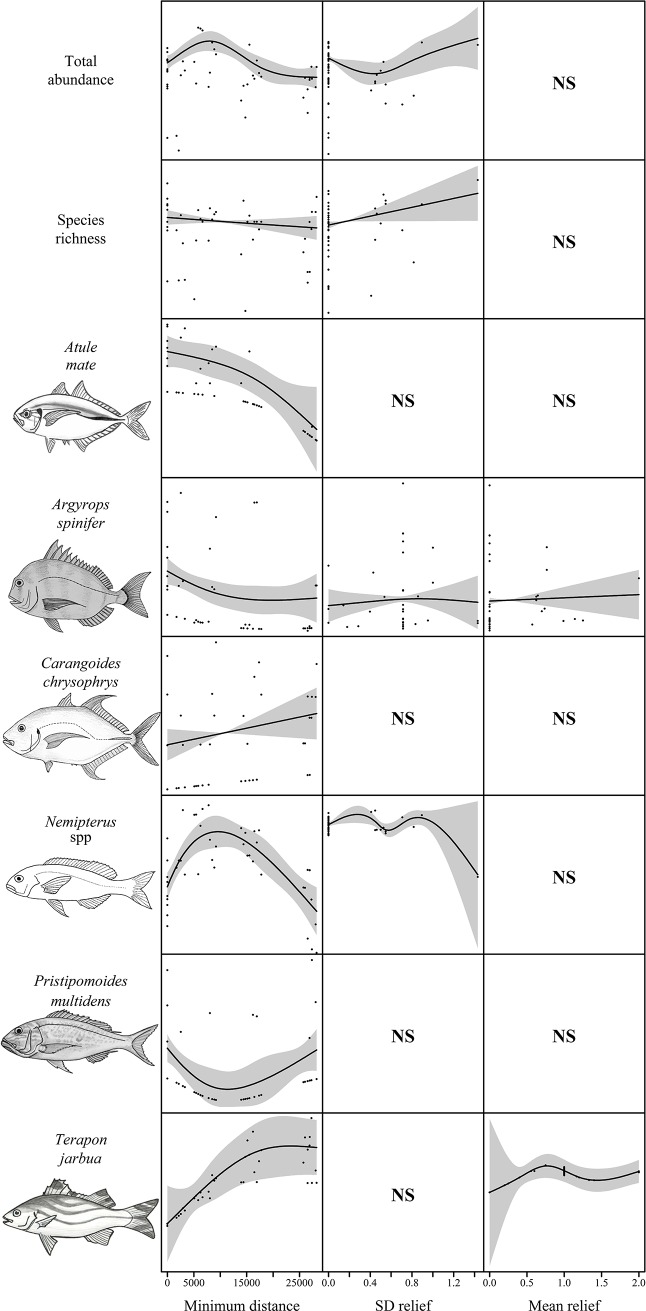
Total fish abundance (sum of MaxN), species richness, and the abundance of key species on deployments in 40–80 m depth relative to the most parsimonious explanatory variables (Table 4). The solid black line represents the estimated smoothing curve and dashed lines represents ±2 x SE of the estimate. Where a Null model was reported as the best model, the second best model was plotted if it was within 2 AIC of the Null model. ‘NS’ indicates the variable was not significant in the model.

Univariate GAMs were produced for total relative abundance and species richness, and those species in Fig 3C. The majority of habitat variables were in such low abundance that no meaningful ecological relationships were evident, therefore only mean relief, standard deviation of relief, and minimum distance to the pipeline were used as predictor variables. As in depths <40 m, minimum distance to the pipeline was present in the best model for species richness and key species (Table 4). Species richness and the abundance of *A*. *spinifer* and *A*. *mate* was higher closer to the pipeline while *T*. *jarbua* increased in abundance with increasing distance from the pipeline (Figs 6 and 7). *Pristipomoides multidens* abundance peaked closer to the pipeline, decreased up to the 12 km from the pipeline then increased again with greater distance from the pipeline (Figs 6 and 7). *Nemipterus* spp. had an inverse relationship to *P*. *multidens* with peaks in abundance at approximately 8 km from the pipeline before decreasing in abundance with increasing distance from the pipeline (Figs 6 and 7).

**Table 4:** Generalised additive models (GAMs) for predicting total abundance, species richness, and the abundance of key species in 40–80 m depth within 2 AIC of the top model.

Dependent variable	eDF	AIC	wAIC	R^2^	Best Model
Total Abundance	6.28	481.41	0.28	0.42	Minimum distance + SD relief
Species Richness	3.03	260.77	0.22	0.15	Minimum distance + SD relief
*Atule mate*	2.91	180.05	0.51	0.24	Minimum distance
*Argyrops spinifer*	5.16	309.28	0.76	0.10	Minimum distance + SD relief + Mean relief
*Carangoides chrysophrys* (1)	1	160.76	0.26	0	Null
(2)	2.00	160.74	0.27	0.04	Minimum distance
*Nemipterus* spp.	7.73	270.69	0.43	0.62	Minimum distance + SD relief
*Pristipomoides multidens*	3.31	59.71	0.96	0.19	Minimum distance
*Terapon jarbua*	5.94	134.35	0.67	0.71	Minimum distance + Mean relief

eDF, estimated degrees of freedom; AIC, Akaike Information Criterion; wAIC, Weighted Akaike Information Criterion. In the case of Carangoides chrysophrys the best model was Null, so the second best model was returned and plotted as it was within 2 AIC.

### Fish-pipeline associations in depths >80 m

In depths >80 m, the five most ubiquitous species on the pipeline were *P*. *multidens* (96% deployments), *A*. *spinifer* (74%), *Nemipterus* spp. (52%), *Seriola dumerili* (greater amberjack, 48%), and *L*. *malabaricus* (44%). The five most ubiquitous species observed off-pipeline were *Nemipterus* spp. (85% deployments), *P*. *multidens* (64%), *Decapterus* sp1 (64%), *A*. *spinifer* (59%), and *L*. *lunaris* (49%) (S1 Table).

**Fig 8:**
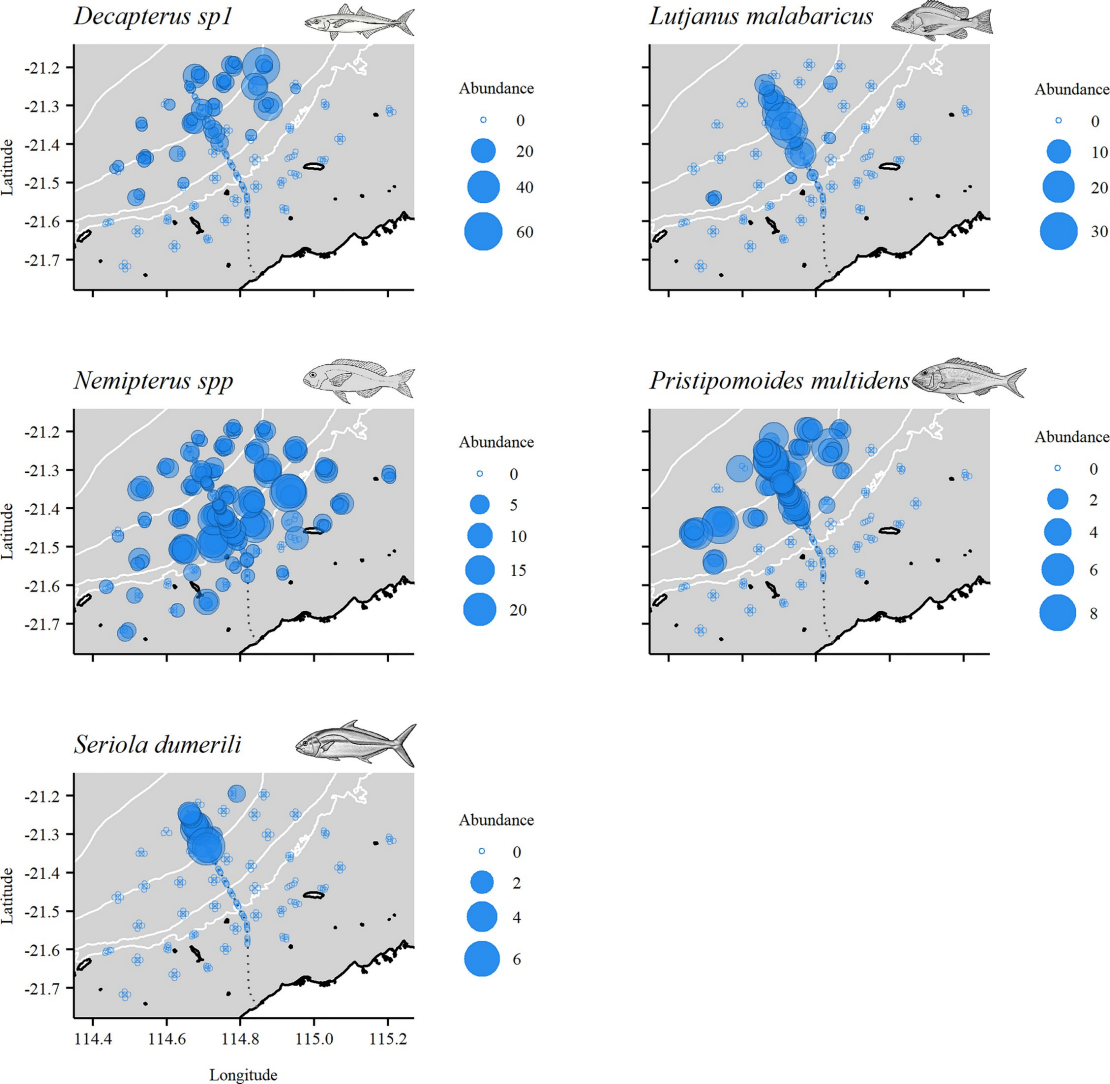
Spatial distribution of the relative abundance of key species in depths >80 m. MaxN bubble sizes reflect abundance, and therefore bubble size may be larger or smaller than those in the legend.

Multivariate analysis of the relative abundance of fish in depths >80 m (S3 Table) showed significant variability among sites (Site(Loc(IvC)): d.f. = 18, 87, MS = 4259.4, Pseudo-F = 3.74, p<0.001), but variation among control locations was not detected over and above this site-level variability (Loc(IvC): p = 0.26). The fish assemblage present along the pipeline (impact site) in depths >80 m was distinct from that observed at control locations at this depth (IvC: p *=* 0.05). In contrast to relative abundance, biomass was only observed to differ among sites in depths >80 m (p<0.001). Fig 3D shows some separation of pipeline and off-pipeline deployments, primarily in PCO1 axis which explained 31% of variation among deployments. Similarly to 40–80 m depth, habitats off the pipeline were predominantly sand (98.27%) and therefore not plotted in Fig 3D. The species responsible for separating pipeline and off-pipeline deployments were *P*. *multidens*, *L*. *malabaricus*, and *S*. *dumerili* associated with pipeline deployments and *Nemipterus* spp. with off-pipeline deployments (Fig 3D). The separation of *Decapterus* sp1 with PCO2 axis likely reflects spatial differences with higher abundance of this species observed east of the pipeline (Fig 8).

**Fig 9:**
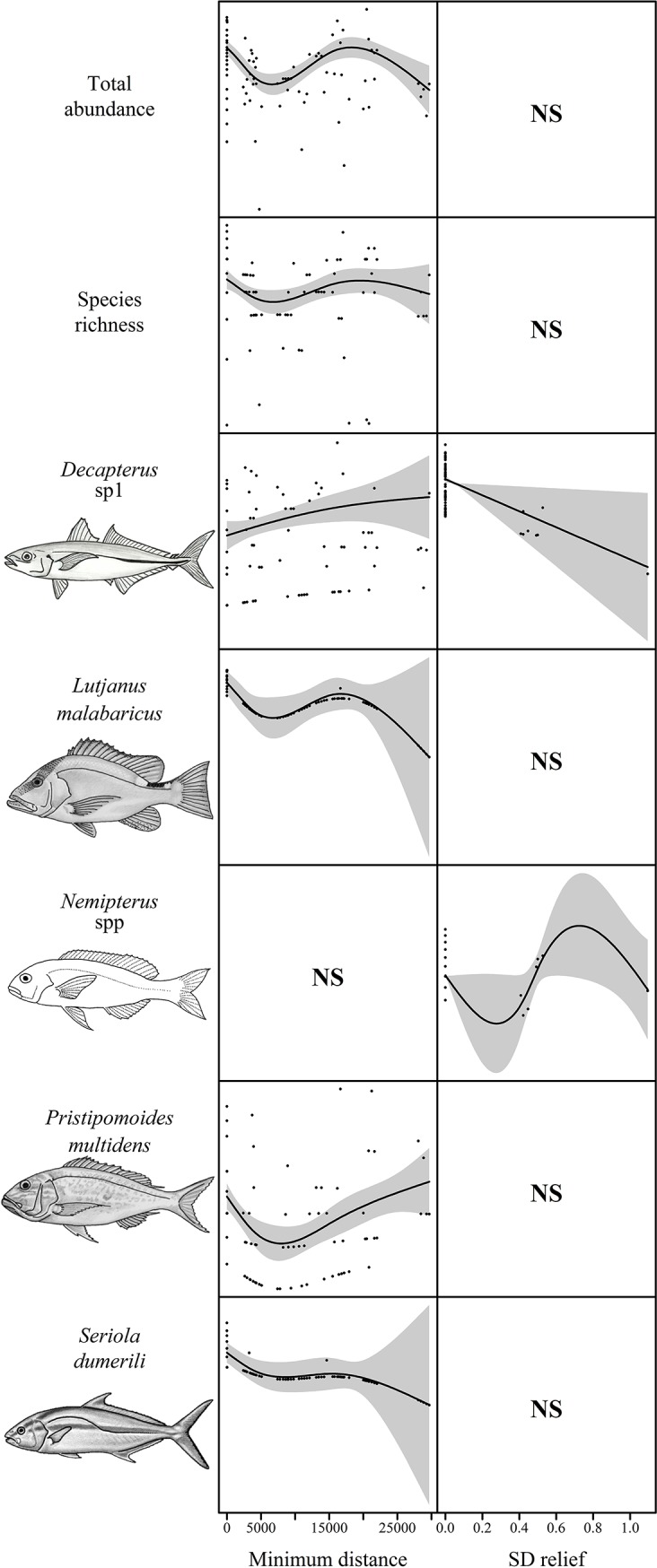
Total fish abundance (sum of MaxN), species richness, and the abundance of key species in depths >80 m relative to the most parsimonious explanatory variables (Table 5). The solid black line represents the estimated smoothing curve and dashed lines represents ±2 x SE of the estimate. Where a Null model was reported a second model was plotted if it was within 2AIC of the Null model. ‘NS’ indicates the variable was not significant in the model.

Univariate GAMs were produced for total relative abundance and species richness, and those species in Fig 3D which best explained the variation in pipeline and off-pipeline deployments. Habitat variables were in such low abundance that no meaningful ecological relationships were evident, therefore only mean relief, SD relief and minimum distance were used as predictor variables. The best model for each species is reported in Table 5 and plotted in Fig 9. Minimum distance was a predictor variable chosen in all but one (*Nemipterus* spp.) of the best models. As with shallower deployments, relationships with minimum distance to the pipeline are negative, reflecting lower residual abundances with increasing distance from the pipeline (Figs 8 and 9). There was, however, a peak in species richness, total abundance, and the relative abundance of *P*. *multidens* and *L*. *malabaricus* at a distance approximately 15 km from the pipeline (Figs 8 and 9). Relatively high abundance of *P*. *multidens* and *L*. *malabaricus* was recorded at a site approximately 15 km south west of the pipeline and may be resulting in the non-linear shape in the GAM plots (Figs 8 and 9).

**Table 5:** Generalised additive models (GAMs) for predicting total abundance, species richness, and the abundance of key species in deep water stereo-BRUV deployments within 2 AIC of the top model.

Dependent variable	eDF	AIC	wAIC	R^2^	Best Model
Total Abundance (1)	4.11	607.87	0.27	0.23	Minimum distance
(2)	5.16	607.13	0.39	0.26	Minimum distance + Mean relief
Species richness (1)	1	355.24	0.19	0	Null
(2)	3.72	353.88	0.37	0.09	Minimum distance
*Decapterus* sp1	3.35	397.83	0.45	0.10	Minimum distance + SD relief
*Lutjanus malabaricus*	3.91	177.54	0.39	0.58	Minimum distance
*Nemipterus* spp.	3.78	339.35	0.76	0.07	SD relief
*Pristipomoides multidens*	3.73	343.59	0.44	0.13	Minimum distance
*Seriola dumerili*	3.45	145.96	0.57	0.51	Minimum distance

eDF, estimated degrees of freedom; AIC, Akaike Information Criterion; wAIC, Weighted Akaike Information Criterion. In the case of Species Richness the best model was Null so the second best model was returned and within 2 AIC.

### Distribution of fish length

**Fig 10:**
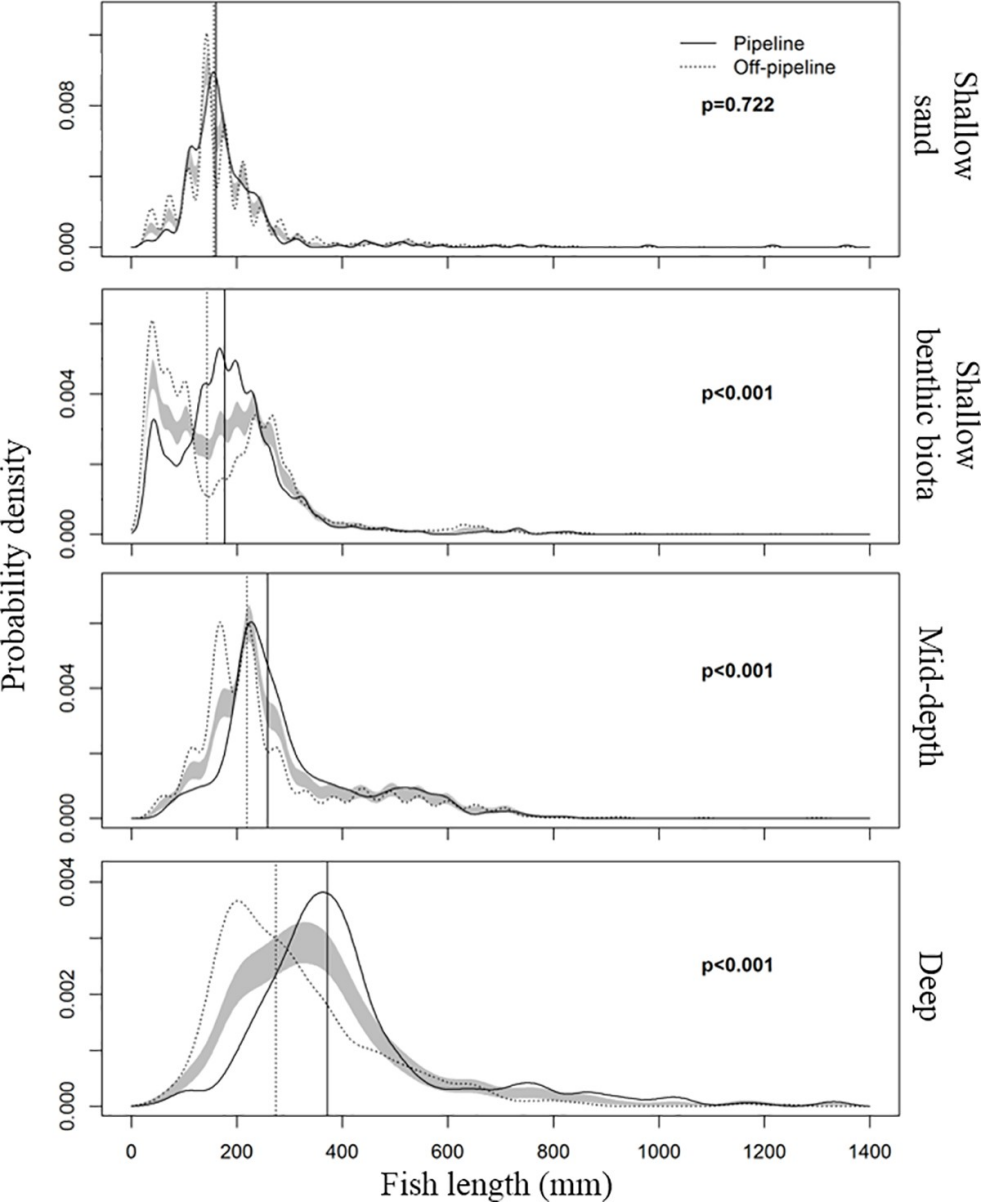
Comparison of the kernel density estimate (KDE) probability function using the mean bandwidth for the lengths of all fish by Location (pipeline and off-pipeline) and depth using raw fish length data. Shallow water deployments are divided into ‘sand’ or ‘benthic biota’ determined by the dominant habitat seen on video footage. Solid and dotted lines present the KDE probability density functions that approximate the length-frequency data of all fish on and off-pipeline, respectively. The shaded band represents 1 SE either side of the null model of no difference between either KDE for each Location. Significance was based on permutation tests of the area between the probability density functions. Vertical lines represent the median fish length from all fish measured on and off-pipeline.

There was no difference in the median fish length recorded in depths <40 m for sand deployments (157.22 mm off and 160.33 mm on the pipeline). However, median fish length was higher on the pipeline for benthic biota deployments in depths <40 m (142.63 mm off and 175.98 mm on the pipeline). As depth increased, the difference in median fish length on and off the pipeline increased (40–80 m: 218.84 mm off and 258.14 mm on; >80 m: 273.56 mm off and 371.25 mm on); larger fish were recorded on the pipeline compared to adjacent areas (Fig 10). The length distribution of all fish differed significantly on and off-pipeline in depths >40 m (p<0.001; Fig 10), but not in <40 m depth (p = 0.16). The shape and location of length probability distribution curves (Fig 10) indicates the presence of a smaller-bodied assemblage off the pipeline and a larger-bodied fish assemblage on the pipeline in depths >40 m. This on-off difference in length distributions was likely primarily driven by the most ubiquitous pipeline and off-pipeline species, *P*. *multidens* and *Nemipterus* spp, respectively.

### Commercially important fish species

**Fig 11:**
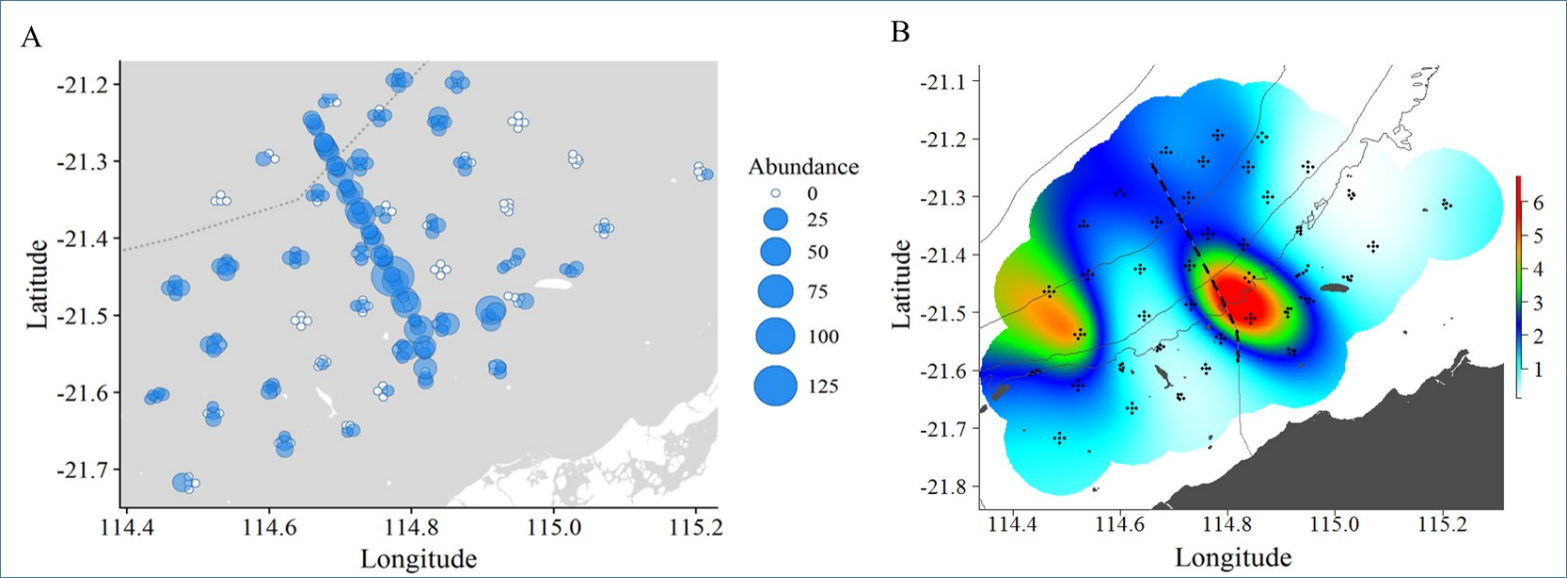
Relative abundance and spatial distribution of biomass of commercial fish species. a) Spatial distribution of the total relative abundance of commercial species (sum of MaxN’s for all commercial species). MaxN bubble sizes reflect abundance, and therefore bubble size may be larger or smaller than those in the legend. b) Smooth spline fits (GAMs) of the total biomass of all measured commercial species. Colour ramp is scaled to the biomass (kg) of all commercial species measured and is predicted by latitude and longitude alone.

**Fig 12:**
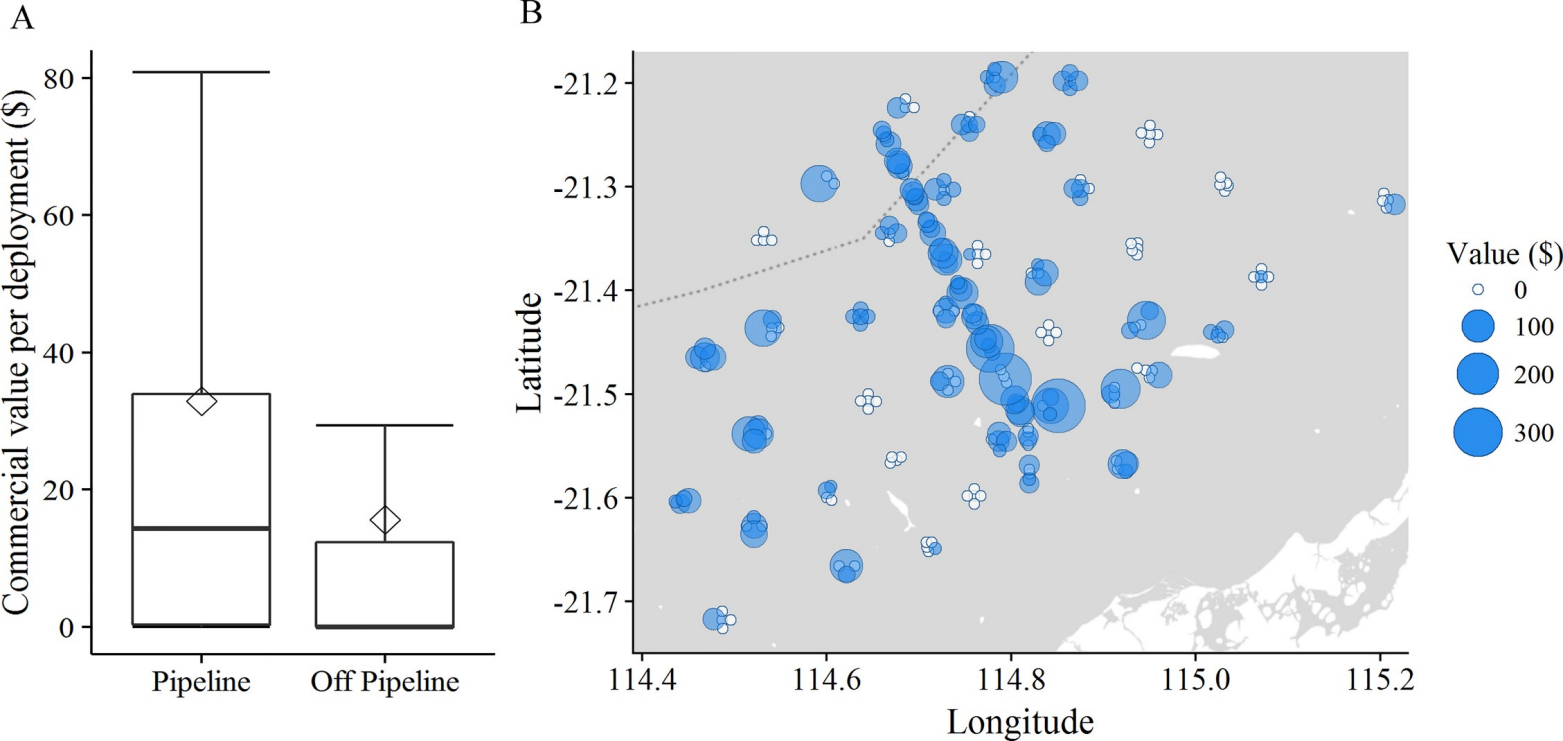
Catch value of commercial species on and off-pipeline. A) Comparisons of the mean (◊) and median (-) catch value recorded on the pipeline and off-pipeline (F(1, 246) = 6.037, p = 0.014). The boxplot show the 95% confidence intervals for the medians, and ranges. B) Spatial distribution of the value of fish recorded in video from each stereo-BRUV deployment.

The total abundance, biomass and catch value of commercial species each showed significant variability among sites (S3 Table; all p<0.01), but variation among control locations was not detected over and above this site-level variability (Loc(IvC): all p >0.6). Commercially fished species present along the pipeline (impact site) were distinct, in terms of abundance, biomass and catch value, from those observed at control locations at the time of this study (IvC: all p < 0.01). Mean total relative abundance was 5x higher, biomass was 1.5x higher and the mean number of species 2.5x higher on the pipeline than off the pipeline (Table 6; Fig 11A). The mean catch value on the pipeline was twice that calculated off-pipeline (Table 6; Fig 12).

**Table 6:** The mean total biomass (kg) of major commercial species and the mean ‘catch value’ per deployment ($) of all major commercial species on and off-pipeline for each depth category and the entire study area.

Depth	Location	Relative abundance (mean ± SE)	Species richness (mean ± SE)	Biomass (kg) (mean ± SE)	Mean catch value per deployment ($AUD mean ± SE)
All	Pipeline	12.98 ± 2.49	2.67 ± 0.24	3.91 ± 0.82	32.87 ± 8.21
	Off Pipeline	2.51 ± 0.36	1.02 ± 0.09	1.82 ± 0.31	15.62 ± 2.97
<40 m	Pipeline	10.25 ± 3.43	0.79 ± 0.12	4.10 ± 1.90	33.21 ± 19.10
	Off Pipeline	2.52 ± 0.64	1.14 ± 0.30	1.67 ± 0.32	15.81 ± 5.10
40–80 m	Pipeline	19.08 ± 9.22	3.39 ± 0.50	5.65 ± 2.08	50.40 ± 22.26
	Off Pipeline	1.83 ± 0.55	1.14 ± 0.30	2.31 ± 0.72	16.21 ± 5.11
>80 m	Pipeline	12.07 ± 2.24	3.15 ± 0.31	2.89 ± 0.60	23.86 ± 4.75
	Off Pipeline	2.89 ± 0.41	1.39 ± 0.13	1.75 ± 0.41	14.98 ± 3.84

Differences in the abundance of commercial species on and off the pipeline was greatest in the 40–80 m depth range (p< 0.01) where the pipeline possessed, on average, 10x more commercial fish than off-pipeline (Table 6). In addition to predicted high abundance of commercial species along the entirety of the pipeline, an area of high abundance is also indicated off the pipeline in Fig 11B. Similarly to abundance, depths of 40–80 m had the greatest difference in mean catch value; $50.40 ± 22.26 SE on the pipeline compared with $16.21 ± 5.11 SE off-pipeline (Table 6; Fig 12).

While the abundance distribution for all commercial species combined shows higher abundance along the pipeline than off-pipeline, the same pattern was not observed for individual commercial species (or genus, families). For example, *Plectropomus* spp. (coral trout) and lethrinids (emperors; considered as important commercial species including *Lethrinus laticaudis* (grass emperor), *Lethrinus nebulosus* (spangled emperor) and *L*. *punctulatus*) were found in <40 m water depth on and off the pipeline in similar abundance while *L*. *malabaricus*, and *Lutjanus russellii* (Moses’ snapper) were encountered more often, and in higher abundance, in depths >40 m on the pipeline (S4 and S5 Figs). *Lutjanus russellii* was recorded on seven pipeline deployments in >80 m depth and not recorded off-pipeline across the entire study area (S4 Fig). Similarly, *L*. *malabaricus* was recorded on six deployments off the pipeline and 16 deployments on the pipeline, predominantly in depths >80 m (S4 Fig).

## Discussion

This study represents the first comprehensive assessment of fish assemblages on a subsea pipeline that spans a depth gradient from 9–140 m with comparison to adjacent natural habitats. The fish assemblage on and around the pipeline changed with respect to depth, distance to the pipeline, and habitats, though these correlates were species-specific. A major finding of the study was that commercial species were found in higher abundance and biomass in association with the pipeline.

Stereo-BRUVs facilitated rapid sampling and provided the opportunity to compare fish assemblages on and off infrastructure, a knowledge gap identified by [15]. Previous work on the north-west shelf by [15] using existing industrial ROV video found a similar fish assemblage to those observed here in 120–140 m, however this study described relatively more *P*. *multidens* and *A*. *spinifer* and fewer *Epinephelus areolatus* (areolate grouper) and *Lutjanus quinquelineatus* (five-lined snapper) in close proximity to the pipeline. Off the southern Californian coast, [14] used a submersible in depths of 95–235 m to compare fish on and off a pipeline. Through use of a submersible they encountered more rockcods and cryptic/site attached species. Differences in assemblages recorded between these studies is likely due to the ROV and submersible being able to sample crypto-benthic species in closer association with pipelines and possibly a flee response of larger predatory species from the noisy and brightly illuminated underwater vehicles, while stereo-BRUVs utilise the attractiveness of bait. Furthermore, the ability of stereo-BRUVs to attract, record and measure predatory species, many of which are commercially important, was an important focus for this study. In both cases, BRUVs and ROVs, the effect of illumination is likely to be species-specific and more important in deeper and/or nocturnal deployments where artificial lighting is much brighter than ambient light. At this point, however, we know little about the exact effect of lights on BRUVS or ROV measurements of fish numbers and this is a subject that requires further research to resolve differences in measurements obtained from different methods.

### Depth and pipeline influences on the fish assemblage

Differences in the fish assemblage including fish length on and off the pipeline, became more evident with increasing depth, and became particularly striking at depths >80 m. This appears to reflect the availability of habitat suitable for fish assemblages within the study area. In depths <40 m, habitats adjacent to the pipeline included reef, macroalgae, and sand and, as such, this nearshore marine environment provides a variety of complex habitats available to support a diverse fish assemblage. A study by [21] examined fish assemblages in natural habitats throughout the nearshore Pilbara region and found that sites in close proximity to the present study had some of the highest soft coral cover and accompanying diversity of fish in the region. The fish assemblage on shallow pipeline BRUVs deployments where biota or the pipeline itself were visible suggest that this on-pipe biota is similar to that in nearby natural ecosystems (Fig 3A). Most fish were, unsurprisingly, reef-associated species such as *P*. *emeryii*, *S*. *fraenatum* and *S*. *monogramma* [39]. This finding supports the suggestion by [57] and [58] that the installation of an artificial structure or reef will only mimic natural adjacent reefs if it possesses similar structural features [59], thus a pipeline will never be equivalent to a limestone reef. The Griffin pipeline, whilst likely not possessing identical fish assemblages to natural reefs in the nearshore area of the Pilbara, does appear to possess a markedly similar fish assemblage in depths <40 m. Differences will more than certainly exist in the cryptic, speciose families’ gobiidae, blenniidae and other crevice dwelling species that may be underrepresented on a pipeline, but this suggestion cannot be confirmed with use of the stereo-BRUVs as this method does not effectively sample these species groups.

Differences between the fish assemblage on the pipeline and those in adjacent natural seabed habitats became more pronounced at greater depths where the availability of complex hard structures for habitat growth is limited on the natural seafloor. In depths >40 m, the fish assemblage on the pipeline was characterised by large bodied, commercially important species including *P*. *multidens*, *L*. *malabaricus*, *L*. *russellii*, *L*. *sebae* and *A*. *spinifer*. These species are commonly associated with structurally complex epibenthic invertebrate communities that include sponges and octocorals [15, 16, 60–64]. These habitats were once common throughout the north-west shelf region, but were reduced by trawl fishing activities between 1959 and 1990 [16]. A change in fish assemblage described by [16] as a consequence of this trawling; an assemblage of high value snappers (Lutjanidae) and emperors (Lethrinidae) switched to smaller bodied lizardfish (Synodontidae) and threadfin bream (Nemipteridae). Our results identify a similar assemblage pattern off-pipeline where *Nemipterus* spp., *S*. *undosquamis*, and *L*. *lunaris* were common (S1 Table). These species are characteristic of muddy and bare sand substrates where they consume infauna and natant crustaceans [65]. The fish assemblage on the pipeline was, in contrast, characterised by high value snappers such as *P*. *multidens* and *L*. *malabaricus*, similar to that described prior to the destruction of epibenthic habitats by historic trawling. Our results suggest that in depths >40 m, the Griffin pipeline possesses a fish assemblage similar to that historically associated with epibenthic communities prior to trawling. With over 2000 km of pipeline on the north-west shelf between Exmouth and Dampier (ENCOM sourced by Woodside Energy Ltd., 2016), research is required to examine and summarise the regional ecological value that pipelines offer.

To understand why certain fish associate with pipelines, it may be necessary to investigate the epibenthic invertebrates colonizing O&G pipelines, and the habitat that they create. Comparable studies examining fish assemblages on pipelines on the north-west shelf found a high diversity of fish at 60–80 m depth and 120–130 m depth using ROV footage [15, 60]. Fish diversity was positively correlated with invertebrates such as sponges and crinoids growing on the pipeline, providing a complex habitat for fish assemblages [15]. Here, however, we were unable to observe close enough the invertebrate biota on the pipeline. When visible, the pipeline did display complex invertebrate colonisation but, where not visible, the assemblage of fish recorded suggested complex, reef-like structures were in close proximity. A combination of on-pipeline video (e.g. by ROV or AUV surveys), to characterise the epibenthic and sclerobiotic fauna, and near/on pipeline BRUVs surveys of fish is required to investigate fish-habitat associations with infrastructure further.

Understanding the length-frequency distribution of fish can give insight into how fish utilise different areas at different stages of their life with important implications for fisheries management [61, 66]. Here, comparing the shape and location of the length-frequency distribution of all fish showed an assemblage comprised of smaller fish off-pipeline and larger fish on the pipeline at depths >40 m. Additionally, deployments with at least 20% visible benthic biota on the pipeline had a significantly larger-bodied assemblage of fish when compared to similar habitats off the pipeline in water <40 m. These results are likely due primarily to the presence of different species on and off the pipeline, as already discussed, rather than a reflection of similar species of differing sizes. Interestingly, no size differences were recorded for the assemblage on and off the pipeline for deployments with 80% or more sand recorded. This could suggest those pipeline associated fish in water depth <40 m are site attached and do not venture far from the pipeline into sandy habitats. It is likely that schools of medium sized (200 mm) *Lutjanus vitta* recorded on pipeline deployments are contributing to the difference in shallow water benthic biota KDEs. McLean et al. [67] recorded *L*. *vitta* on 66% of O&G wellhead infrastructure sampled in 85–135 m water depth, however it is unknown if their presence is specifically associated with this infrastructure or a random schooling event. Investigation of species-specific length-frequency distributions did not reveal any clear ontogenetic shifts with depth, location or habitat, however this was not unexpected given the relatively low numbers of individuals for most species across all factors. *Pristipomoides multidens* and *A*. *spinifer* were found in higher abundance in depths of 40–80 m and >80 m, both on and off-pipeline, but possessed similar length distributions. The biology of *P*. *multidens* is poorly understood and habitats that juveniles reside in are unknown [63]. Studies that examine fish assemblage size structure on multiple onshore-offshore pipelines, or on a single pipeline over time, would provide further insights into how fish interact with infrastructure at different life stages.

Several IUCN red list species were recorded in this study (S1 and S2 Tables) [68]. A critically endangered green sawfish (*Pristis zijsron*) was observed (see Fig 1) on a stereo-BRUV deployed ~1.5 m from the pipeline. The length of this individual (ca. 3.76 m) suggests that it is approximately 8–10 years of age [69]. Additional IUCN endangered species included the scalloped hammerhead (*Sphyrna lewini*), great hammerhead shark (*Sphyrna mokarran*), and zebra shark (*Stegastoma fasciatum*). A further ten vulnerable and 12 near threatened species were observed in this study. Given the population status of these important and vulnerable species, there is much species-specific research being conducted into the movements and life history stages [70, 71]. The offshore O&G industry could further support this research by: 1) reporting sightings of these species in industry ROV video footage obtained on structures, and 2) utilise acoustic tracking receivers on ROVs (if acoustic interference from the ROV allows) or on subsea infrastructure to detect tagged individuals when they are nearby. This information would provide scientists with valuable movement information on these species and further knowledge of the value of subsea infrastructure for endangered species.

### Commercial fish and fisheries

Understanding how commercial species may interact with subsea infrastructure, and if this interaction is different from that with the adjacent natural habitat, is important for the current and future management of fisheries on the north-west shelf and for informing decommissioning planning. Commercially targeted fish species showed a stronger association with the pipeline relative to the total fish assemblage, with greater variety and higher abundance and biomass of commercial species found on the pipeline compared to the surrounding areas. In addition, fish on the pipeline were larger than those off-pipeline (Table 6). The increased abundance and biomass of commercially important species found on the pipeline was due to the high abundance of snappers, specifically *L*. *malabaricus*, *L*. *sebae*, *L*. *russellii* and *P*. *multidens*. In shallower waters (<40 m) a high abundance of commercial species was also observed off the pipeline, primarily of lethrinids (emperor) and of commercially valuable *Plectropomus* spp. and *Epinephelus multinotatus* (Rankin cod). Natural reef and macroalgal habitat surrounding many of the islands in the area is ideal for lethrinids [21] and this factor is likely contributing to more similar distribution of commercial important species in shallow waters on and off-pipeline.

A significantly greater abundance and biomass of commercial species on the pipeline translates to the catch value of commercial species recorded being nearly twice as high as that recorded off-pipeline. This difference is driven by lutjanid species, especially *L*. *sebae* and *L*. *malabaricus* (S4 Fig; $11.62 and $5.36AUD per kilogram of whole fish respectively) which were recorded in large numbers on the pipeline and almost nowhere else. It is possible that *L*. *malabaricus* is particularly attracted to the habitat around offshore infrastructure as [9] and [67] also noted *L*. *malabaricus* on well head infrastructure in the north-west shelf region of WA. Trap fishers target pipelines and other infrastructure on the north-west shelf to obtain higher catches, although a knowledge gap exists regarding the amount of effort expended on infrastructure. Historical data from vessel monitoring systems (VMS) was used to understand how trawl fishers may be targeting pipeline infrastructure in the North Sea [72]. It is a licensing requirement that VMS are installed on trap fishing boats on the north-west shelf making a similar study feasible, should the location of all infrastructure be available. It is evident that the Griffin pipeline holds high numbers of commercially valuable fish species, however further investigation is needed to ascertain the extent to which commercial fishers target pipelines such as this one.

Commercial fish value in depths <40 m was driven by the abundance of lethrinid species, but also by the presence of high value *Plectropomus spp*. ($15.09AUD per kilogram of whole fish) and *E*. *multinotatus* ($8.43AUD per kilogram of whole fish). These species were recorded on the pipeline in shallower waters, but were supplemented by lutjanids which appeared regularly in association with the pipeline at this depth. The distribution of the valuable and iconic coral trout (*Plectropomus* spp.) and emperors (lethrinids; namely *L*. *laticaudis*, *L*. *nebulosus* and *L*. *punctulatus*) in the shallows occurred both on and off-pipeline. This is likely due to their preferred habitat (see [73]) being relatively more available and well distributed throughout the shallows, meaning the pipeline is not providing unique habitat benefits. These results highlight that the influence of the pipeline on commercial species can be both species-specific and depth-dependent. Understanding how each commercial species interacts with subsea infrastructure across a depth range is essential to inform fisheries management and as decommissioning options are considered. Furthermore, considering 74% of boat-based recreational fishing occurs in nearshore or inshore waters in the region [74], one must also consider how recreational fishers are impacting fish on and around subsea infrastructure in the nearshore to fully understand the value of pipeline infrastructure on targeted fish. *Scomberomorus* spp., although not in the top 15 major and iconic commercial species in the North Coast Bioregion, are within the Mackerel Managed Fishery (MMF) and considered an important fishery state-wide. They are also targeted by recreational fisheries and *Scomberomorus commerson* (Spanish mackerel) is in the top five of retained species by number in the North Coast Bioregion and Gascoyne Bioregion [74]. In this study, the distribution of *Scomberomorus* spp. was defined by depth, with higher abundance of fish recorded and predicted in depths <40 m (see S5 Fig), however no difference in abundance was seen between deployments on and off-pipeline. These results further emphasise the importance of understanding the ecology of important and valuable commercial and recreational fish, and how they may interact with subsea infrastructure in shallow as well as deep waters.

### Implications for management and future research

Decommissioning subsea infrastructure will pose a significant and increasing challenge globally in the coming decades, particularly in locations such as the north-west shelf of Australia and Bass Strait where there is a relatively high concentration of infrastructure and limited local decommissioning experience and capability. Decisions regarding the fate of infrastructure after decommissioning should be made on a case-by-case cost-benefit analysis basis incorporating sufficient scientific knowledge to predict impacts, including ecological, with an acceptable degree of uncertainty. In Australia, offshore petroleum regulations require consideration of the full removal of offshore infrastructure at decommissioning as the ‘base-case’. This requirement is, however, subject to other provisions of offshore petroleum regulations, where options other than complete removal may be considered, provided the alternative decommissioning approach demonstrably delivers equal or better environmental outcomes compared to complete removal of subsea facilities, and that the approach complies with all legislative and regulatory obligations. Removal of offshore infrastructure involves significant cost and risk and may disrupt or destroy marine ecosystems that have developed on these structures over decades of operational life. This study shows that the Griffin pipeline possesses diverse and abundant fish life, including valuable commercially targeted fish species, which should be considered in decommissioning scenarios. Further research into the influence of infrastructure in marine ecosystems of the north-west shelf is required to guide future development and inform decommissioning decisions. In addition, when individual pipelines or other subsea infrastructure is being consider for removal, BRUVs and/or ROV video surveys of each asset is suggested to better inform decision makers.

## Conclusions

This study is the first to use BRUVs to report on the way in which a subsea pipeline affects fish communities along a depth gradient, and as such represents a valuable perspective on the ecological importance of this form of subsea infrastructure. We recommend that the ecological value of pipelines be placed in a wider context beyond investigating influences on fish communities. For example, research on the impacts of pipelines on ecosystem biogeochemistry could reveal if they act as ‘productivity hotspots’ leading to an export of productivity to or from adjacent ecosystems, in a manner similar to deep-water corals or submarine canyons [74]. It is clear that structures such as pipelines are often heavily laden with sclerobiont biofouling organisms, but the relationship of this fauna with the natural benthic fauna that was endemic to the north-west shelf region prior to trawling in the last century is unknown, as is the potential for biofouling organisms to recolonise the currently relatively depauperate sandy habitat of the region, and its ability to re-establish the previous commercial fish populations. It would also be beneficial to investigate further the influence of pipelines on key aspects of fish behaviour. For example, [7] described the feeding behaviour of Australian fur seals along anthropogenic seafloor structures in Bass Strait, suggesting this was a regular behaviour and foraging area. Do foraging behaviours of predatory fish species change similarly on pipelines compared with adjacent habitat? Do individuals move along pipelines, across depth gradients, with ontogenetic shifts? Additionally, do juveniles remain associated with a pipeline throughout their life history, or move off that pipeline into adjacent habitats? Finally, the socio-economic costs and benefits associated with subsea infrastructure could be better characterised. Though the value of the pipeline for commercial fisheries is beginning to be demonstrated, the value to recreational fisheries is less well understood, yet will be an important component of the cost-benefit analysis when determining the fate of subsea infrastructure.

## Supporting information

S1 Table The abundance and commonality (% deployments) of all bony fish species (alphabetical by order then family and then genus) observed on the Griffin pipeline deployments and those in the surrounding natural environment (off pipeline). Species that appear as near threatened on the IUCN Red List are identified with *NT*. Species listed as “sp10” were not identifiable to species level but are included as they were different species to all others listed. Species with a “sp#” were unidentifiable during the course of this study, but thought to be identified in the future.
(DOCX)

S2 Table The abundance and commonality (% deployments) of all cartilaginous fish species (alphabetical by order then family and then genus) observed on the Griffin pipeline and those in the surrounding natural environment (off pipeline). Species that appear as critically endangered (*CE*), endangered (*EN*), vulnerable (*VU*), or near threatened (*NT)* on the IUCN Red List are identified. Species listed as “sp10” were not identifiable to species level but are included as they were different species to all others listed.
(DOCX)

S3 Table Results of PERMANOVA analyses on the fish assemblage relative abundance and biomass (multivariate analyses; fourth-root transformed data, Bray Curtis dissimilarities) and on the total abundance and biomass of commercial species (univariate analyses; fourth-root transformed, Euclidean Distance dissimiliarities). The results of subsequent depth range analyses results are also presented. Abundance results are in blue and biomass in orange.
(DOCX)

S4 Table Best generalised additive models (GAMs) for predicting total abundance, species richness, and the abundance of key species identified in Fig 2. Key species are divided into <40m 40-80m and >80 m depth categories depending on the direction of the vectors in the CAP. eDF, estimated degrees of freedom; AIC, Akaike Information Criterion; wAIC, Weighted Akaike Information Criterion.
(DOCX)

S5 Table Summary of the commercial catches and the relative contribution (% composition) of each of the major or iconic species taken within the Pilbara and Kimberley sectors of the North Coast Bioregion in 2015 (Fletcher et al. 2017). (DOCX)

S1 Fig Spatial distribution of the relative abundance of key species. MaxN bubble sizes reflect exact abundance, and therefore bubble size may be larger or smaller than those in the legend’s categories.
(TIF)

S2 Fig Total fish abundance, species richness, and the abundance of key species relative to the most parsimonious explanatory variables (Table 4). The solid black line represents the estimated smoothing curve and dashed lines represents ±2 x SE of the estimate.
(TIF)

S3 Fig Smooth spline fits (GAMs) of the predicted abundance of key species; *C. caeruleopinnatus*, and *Nemipterus* spp. (40–80 m) and *A. spinifer*, *Decapterus* sp1 and *P. multidens* (>80 m). Colour ramp represents the abundance predicted by latitude and longitude alone. Heatmaps of other common and interesting species are presented in Appendix 4.
(TIFF)

S4 Fig Spatial distribution of the relative abundance of commercial species found throughout the study area, including *L. sebae, L. malabaricus, L erythropterus, L. vitta, L. russellii, P. multidens, L. nebulosus, L. punctulatus* and *L laticaudis*. MaxN bubble sizes reflect actual abundance, and therefore bubble size may be larger or smaller than those in the legend.
(TIFF)

S5 Fig Spatial distribution of the relative abundance of commercial species found throughout the study area, including *G. grandoculis, Scopmberomberous* spp, *E. multinotatus, Plectropomus* spp, and *A. spinifer*. MaxN bubble sizes reflect actual abundance, and therefore bubble size may be larger or smaller than those in the legend.
(TIFF)
