# Supplementary material for: The influence of depth and a subsea pipeline on fish assemblages and commercially fished species
Source: PLoS One. 2018 Nov 26;13(11):e0207703. doi: 10.1371/journal.pone.0207703 (PMC6257935; doi:10.1371/journal.pone.0207703)
Supplement: S1 Table — Species that appear as near threatened on the IUCN Red List are identified with NT. Species listed as “sp10” were not identifiable to species level but are included as they were different species to all others listed. Species with a “sp#” were unidentifiable during the course of this study, but thought to be identified in the future. (DOCX) [file pone.0207703.s001.docx]

S-Table 1: The abundance and commonality (% deployments) of all bony fish species (alphabetical by Order then family and then genus) observed on the Griffin pipeline deployments and those in the surrounding natural environment (Off pipeline). Species that appear as near threatened on the IUCN Red List are identified with *NT*. Species listed as “sp10” were not identifiable to species level but are included as they were different species to all others listed. Species with a “sp#” were unidentifiable during the course of this study, but thought to be identified in the future.

| ***Order*/**Family | Species | Common Name | Total Abundance | Commonality (%) | | Mean Abundance ±SE | |
| --- | --- | --- | --- | --- | --- | --- | --- |
|  |  |  |  | Pipeline | Off-pipeline | Pipeline | Off-pipeline |
| ***Anguilliformes*** |  |  |  |  |  |  |  |
| Congridae | *Conger cinereus* | Conger eel | 2 | 1.67 | 0.51 | 0.02 ±0.02 | 0.01 ±0.01 |
| Muraenidae | *Gymnothorax flavimarginatus* | Yellow-edged moray | 1 |  | 0.51 |  | 0.01 ±0.01 |
|  | *Gymnothorax rueppellii* | Banded moray | 1 |  | 0.51 |  | 0.01 ±0.01 |
|  | *Gymnothorax thrysoideus* | Grayface moray | 2 |  | 1.03 |  | 0.01 ±0.01 |
| ***Aulopiformes*** |  |  |  |  |  |  |  |
| Synodontidae | *Saurida undosquamis* | Brushtooth lizardfish | 64 | 8.33 | 26.15 | 0.10 ±0.05 | 0.30 ±0.04 |
|  | *Synodus dermatogenys* | Banded lizardfish | 1 |  | 0.51 |  | 0.01 ±0.01 |
|  | *Trachinocephalus myops* | Painted grinner | 1 |  | 0.51 |  | 0.01 ±0.01 |
| ***Elopiformes*** |  |  |  |  |  |  |  |
| Elopidae | *Elops hawaiensis* | Giant Herring | 9 |  | 1.54 |  | 0.05 ±0.03 |
| ***Clupeiformes*** |  |  |  |  |  |  |  |
| Clupeidae | *Clupeidae spp* | Sprats | 2000 | 3.33 | 3.08 | 5.73 ±4.15 | 8.49 ±3.60 |
| ***Perciformes*** |  |  |  |  |  |  |  |
| Acanthuridae | *Acanthurus grammoptilus* | Ring-tailed surgeonfish | 23 | 5.00 | 4.62 | 0.08 ±0.05 | 0.09 ±0.04 |
|  | *Acanthurus olivaceus* | Orangeblotch surgeonfish | 5 |  | 1.03 |  | 0.03 ±0.02 |
|  | *Naso annulatus* | Ringtail unicornfish | 51 | 5.00 | 5.13 | 0.13 ±0.08 | 0.22 ±0.12 |
|  | *Naso fageni* | Horseface unicornfish | 1 |  | 0.51 |  | 0.01 ±0.01 |
| Apogonidae | *Apogonidae spp* | Cardinal fish | 277 |  | 3.08 |  | 1.42 ±1.26 |
| Blenniidae | *Ecsenius bicolor* | Bicolor combtooth blenny | 3 |  | 1.54 |  | 0.02 ±0.01 |
|  | *Plagiotremus rhinorhynchos* | Bluestriped fangblenny | 2 |  | 1.03 |  | 0.01 ±0.01 |
|  | *Plagiotremus tapeinosoma* | Piano fangblenny | 10 |  | 3.08 |  | 0.05 ±0.02 |
|  | *Blenniidae spp* | blenny | 1 |  | 0.51 |  | 0.01 ±0.01 |
| Caesionidae | *Caesio cuning* | Yellowtail fusilier | 64 |  | 0.51 |  | 0.33 ±0.33 |
|  | *Pterocaesio chrysozona* | Yellowband fusilier | 184 | 1.67 | 1.54 | 0.52 ±0.52 | 0.78 ±0.61 |
|  | *Pterocaesio digramma* | Doubleline fusilier | 468 | 8.33 | 2.05 | 3.85 ±2.17 | 1.22 ±0.76 |
| Carangidae | *Alepes vari* | Herring scad | 2 |  | 0.51 |  | 0.01 ±0.01 |
|  | *Atule mate* | Yellowtail scad | 450 | 11.67 | 13.33 | 1.77 ±0.87 | 1.76 ±0.62 |
|  | *Carangoides caeruleopinnatus* | Onion trevally | 172 | 30.00 | 30.77 | 0.73 ±0.18 | 0.66 ±0.10 |
|  | *Carangoides chrysophrys* | Longnose trevally | 106 | 11.67 | 23.08 | 0.38 ±0.17 | 0.43 ±0.07 |
|  | *Carangoides fulvoguttatus* | Gold-spotted trevally | 112 | 1.67 | 8.21 | 0.02 ±0.02 | 0.57 ±0.22 |
|  | *Carangoides gymnostethus* | Bludger trevally | 153 | 11.67 | 14.36 | 0.28 ±0.12 | 0.70 ±0.23 |
|  | *Carangoides hedlandensis* | Bunmpnose trevally | 12 |  | 3.08 |  | 0.06 ±0.03 |
|  | *Carangoides orthogrammus* | Thicklip trevally | 1 |  | 0.51 |  | 0.01 ±0.01 |
|  | *Caranx ignobilis* | Giant trevally | 28 | 5.00 | 5.64 | 0.05 ±0.03 | 0.13 ±0.06 |
|  | *Caranx papuensis* | Brassy trevally | 1 | 1.67 |  | 0.02 ±0.02 |  |
|  | *Decapterus sp1* | Mackerel scad | 380 | 15.00 | 24.62 | 0.90 ±0.37 | 1.67 ±0.41 |
|  | *Gnathanodon speciosus* | Golden trevally | 479 | 8.33 | 14.36 | 0.52 ±0.26 | 2.30 ±0.65 |
|  | *Pantolabus radiatus* | Fringefin trevally | 1 |  | 0.51 |  | 0.01 ±0.01 |
|  | *Parastromateus niger* | Black Pomfret | 4 | 1.67 | 0.51 | 0.05 ±0.05 | 0.01 ±0.01 |
|  | *Scomberoides commersonnianus* | Giant queenfish | 1 |  | 0.51 |  | 0.01 ±0.01 |
|  | *Scomberoides lysan* | Lesser queenfish | 3 |  | 0.51 |  | 0.02 ±0.02 |
|  | *Selaroides leptolepis* | Yellowstripe Scad | 2304 | 18.33 | 26.67 | 3.97 ±1.47 | 10.59 ±2.11 |
|  | *Seriola dumerili* | Amberjack | 44 | 21.67 | 1.03 | 0.70 ±0.20 | 0.01 ±0.01 |
|  | *Seriola rivoliana* | Highfin amberjack | 2 |  | 0.51 |  | 0.01 ±0.01 |
|  | *Seriolina nigrofasciata* | Blackbanded amberjack | 15 | 1.67 | 6.67 | 0.02 ±0.02 | 0.07 ±0.02 |
|  | *Trachurus sp1* | Scad | 125 | 3.33 | 2.05 | 0.20 ±0.15 | 0.58 ±0.40 |
|  | *Uraspis secunda* | Cottonmouth trevally | 23 | 1.67 | 0.51 | 0.08 ±0.08 | 0.09 ±0.09 |
|  | *Carangidae sp1* | Trevally species | 6 |  | 0.51 |  | 0.03 ±0.03 |
|  | *Carangidae sp10* | Trevally species | 1 |  | 0.51 |  | 0.01 ±0.01 |
| Chaetodontidae | *Chaetodon assarius* | Western butterflyfish | 34 | 5.00 | 5.13 | 0.07 ±0.04 | 0.15 ±0.10 |
|  | *Chaetodon aureofasciatus* | Goldstripe butterflyfish | 11 | 1.67 | 2.05 | 0.08 ±0.08 | 0.03 ±0.02 |
|  | *Chaetodon auriga* | Threadfin butterflyfish | 4 | 1.67 | 1.03 | 0.02 ±0.02 | 0.02 ±0.01 |
|  | *Chaetodon kleinii* | Klein’s butterflyfish | 2 |  | 0.51 |  | 0.01 ±0.01 |
|  | *Chaetodon plebeius* | Bluespot butterflyfish | 4 |  | 1.03 |  | 0.02 ±0.01 |
|  | *Chelmon marginalis* | Margined coralfish | 28 | 3.33 | 6.15 | 0.07 ±0.05 | 0.12 ±0.03 |
|  | *Coradion altivelis* | Highfin coralfish | 1 |  | 0.51 |  | 0.01 ±0.01 |
|  | *Coradion chrysozonus* | Orangebaned coralfish | 4 |  | 1.54 |  | 0.02 ±0.01 |
|  | *Heniochus acuminatus* | Longfin bannerfish | 10 | 1.67 | 0.51 | 0.02 ±0.02 | 0.05 ±0.05 |
|  | *Parachaetodon ocellatus* | Ocellate butteflyfish | 6 | 3.33 | 0.51 | 0.08 ±0.06 | 0.01 ±0.01 |
| Echeneidae | *Echeneis naucrates* | Sharksucker | 95 | 20.00 | 18.97 | 0.43 ±0.15 | 0.35 ±0.08 |
| Epinephelidae | *Cephalopholis boenak* | Brown-banded rockcod | 1 |  | 0.51 |  | 0.01 ±0.01 |
|  | *Cephalopholis sonnerati* | Tomato rockcod | 2 |  | 0.51 |  | 0.01 ±0.01 |
|  | *Diploprion bifasciatum* | Barred soapfish | 1 |  | 0.51 |  | 0.01 ±0.01 |
|  | *Epinephelus areolatus* | Areolate grouper | 18 | 11.67 | 2.56 | 0.18 ±0.07 | 0.04 ±0.02 |
|  | *Epinephelus bilobatus* | Frostback rockcod | 46 | 8.33 | 7.69 | 0.30 ±0.15 | 0.14 ±0.04 |
|  | *Epinephelus coioides ^NT^* | Orange-spotted grouper | 7 | 3.33 | 2.56 | 0.03 ±0.02 | 0.03 ±0.01 |
|  | *Epinephelus corallicola* | Coral grouper | 2 | 1.67 |  | 0.03 ±0.03 |  |
|  | *Epinephelus fasciatus* | Blacktip grouper | 48 | 3.33 | 7.69 | 0.08 ±0.07 | 0.22 ±0.07 |
|  | *Epinephelus multinotatus* | Rankin cod | 8 | 3.33 | 1.54 | 0.03 ±0.02 | 0.03 ±0.02 |
|  | *Epinephelus polyphekadion ^NT^* | Camouflage grouper | 1 | 1.67 |  | 0.02 ±0.02 |  |
|  | *Epinephelus quoyanus* | Longfin rockcod | 2 | 1.67 | 0.51 | 0.02 ±0.02 | 0.01 ±0.01 |
|  | *Epinephelus rivulatus* | Halfmoon grouper | 5 | 1.67 | 1.54 | 0.02 ±0.02 | 0.02 ±0.01 |
|  | *Plectropomus spp* | Coral trout | 31 | 8.33 | 7.18 | 0.15 ±0.07 | 0.11 ±0.03 |
| Epippidae | *Platax batavianus* | Humphead batfish | 9 |  | 3.08 |  | 0.05 ±0.02 |
|  | *Platax orbicularis* | Round batfish | 1 |  | 0.51 |  | 0.01 ±0.01 |
| Gerreidae | *Gerreidae sp10* | Silverbiddy | 158 |  | 0.51 |  | 0.81 ±0.81 |
| Glaucosomatidae | *Glaucosoma buergeri* | Northern pearl perch | 14 | 10.00 |  | 0.23 ±0.12 |  |
|  | *Glaucosoma magnificum* | Threadfin pearl perch | 72 | 1.67 |  | 1.20 ±1.20 |  |
| Haemullidae | *Hapalogenys dampieriensis* | Australian striped velvetchin | 1 | 1.67 |  | 0.02 ±0.02 |  |
|  | *Diagramma pictum* | Painted sweetlips | 18 | 15.00 | 3.08 | 0.18 ±0.07 | 0.04 ±0.02 |
|  | *Plectorhinchus gibbosus* | Brown sweetlips | 1 | 1.67 |  | 0.02 ±0.02 |  |
|  | *Pomadasys kaakan* | Barred javelin | 2 | 1.67 | 0.51 | 0.02 ±0.02 | 0.01 ±0.01 |
| Kyposidae | *Kyphosus bigibbus* | Grey drummer | 1 |  | 0.51 |  | 0.01 ±0.01 |
| Labridae | *Anampses lennardi* | Blue and yellow wrasse | 3 | 1.67 | 1.03 | 0.02 ±0.02 | 0.01 ±0.01 |
|  | *Bodianus bilunulatus* | Saddleback pigfish | 11 |  | 3.59 |  | 0.06 ±0.02 |
|  | *Bodianus solatus* | Sunburnt pigfish | 4 | 3.33 | 0.51 | 0.05 ±0.04 | 0.01 ±0.01 |
|  | *Cheilinus chlorourus* | Floral wrasse | 2 |  | 1.03 |  | 0.01 ±0.01 |
|  | *Cheilio inermis* | Sharpnose wrasse | 1 |  | 0.51 |  | 0.01 ±0.01 |
|  | *Choerodon cauteroma* | Bluespotted tuskfish | 100 | 26.67 | 17.95 | 0.48 ±0.12 | 0.36 ±0.07 |
|  | *Choerodon cephalotes* | Purple tuskfish | 29 | 3.33 | 8.21 | 0.12 ±0.09 | 0.11 ±0.04 |
|  | *Choerodon cyanodus* | Blue tuskfish | 19 | 6.67 | 5.64 | 0.08 ±0.04 | 0.07 ±0.02 |
|  | *Choerodon schoenleinii ^NT^* | Blackspot tuskfish | 12 | 3.33 | 4.62 | 0.05 ±0.04 | 0.05 ±0.02 |
|  | *Choerodon vitta* | Redstripe tuskfish | 19 |  | 5.13 |  | 0.10 ±0.04 |
|  | *Cirrhilabrus temminckii* | Peacock wrasse | 73 |  | 3.08 |  | 0.37 ±0.29 |
|  | *Coris caudimacula* | Spot-tail wrasse | 115 | 1.67 | 13.33 | 0.03 ±0.03 | 0.58 ±0.15 |
|  | *Coris dorsomacula* | Pinklined wrasse | 2 |  | 0.51 |  | 0.01 ±0.01 |
|  | *Coris pictoides* | Pixy wrasse | 4 |  | 1.54 |  | 0.02 ±0.01 |
|  | *Halichoeres hortulanus* | Checkerboard wrasse | 1 |  | 0.51 |  | 0.01 ±0.01 |
|  | *Halichoeres nebulosus* | Cloud wrasse | 15 |  | 4.62 |  | 0.08 ±0.03 |
|  | *Halichoeres nigrescens* | Bubblefin wrasse | 5 |  | 0.51 |  | 0.03 ±0.03 |
|  | *Halichoeres trimaculatus* | Threespot wrasse | 1 | 1.67 |  | 0.02 ±0.02 |  |
|  | *Hologymnosus annulatus* | Ring wrasse | 2 |  | 1.03 |  | 0.01 ±0.01 |
|  | *Iniistius pavo* | Blue razorfish | 9 |  | 4.10 |  | 0.05 ±0.02 |
|  | *Labroides dimidiatus* | Common cleanerfish | 69 | 8.33 | 13.33 | 0.23 ±0.10 | 0.28 ±0.06 |
|  | *Leptojulis chrysotaenia* | Ochreband wrasse | 5 |  | 2.05 |  | 0.03 ±0.01 |
|  | *Leptojulis cyanopleura* | Shoulderspot wrasse | 60 | 5.00 | 6.67 | 0.15 ±0.09 | 0.26 ±0.10 |
|  | *Macropharyngodon negrosensis* | Black leopard wrasse | 4 |  | 0.51 |  | 0.02 ±0.02 |
|  | *Macropharyngodon ornatus* | Ornate leopard wrasse | 1 |  | 0.51 |  | 0.01 ±0.01 |
|  | *Pseudocheilinus evanidus* | Pinstripe wrasse | 1 |  | 0.51 |  | 0.01 ±0.01 |
|  | *Pseudojuloides severnsi* | Severn’s wrasse | 2 |  | 0.51 |  | 0.01 ±0.01 |
|  | *Stethojulis bandanensis* | Redspot wrasse | 1 |  | 0.51 |  | 0.01 ±0.01 |
|  | *Stethojulis interrupta* | Brokenline wrasse | 8 |  | 2.05 |  | 0.04 ±0.02 |
|  | *Stethojulis strigiventer* | Threeline wrasse | 1 |  | 0.51 |  | 0.01 ±0.01 |
|  | *Thalassoma lunare* | Moon wrasse | 24 | 1.67 | 3.08 | 0.18 ±0.18 | 0.07 ±0.03 |
| Labridae (scarinae) | *Chlorurus microrhinos* | Steephead parrotfish | 2 |  | 1.03 |  | 0.01 ±0.01 |
|  | *Scarus chameleon* | Chameleon parrotfish | 1 |  | 0.51 |  | 0.01 ±0.01 |
|  | *Scarus ghobban* | Blue-barred parrotfish | 26 | 6.67 | 5.13 | 0.08 ±0.04 | 0.11 ±0.04 |
|  | *Scarus sp2* | Parrotfish | 11 |  | 0.51 |  | 0.06 ±0.06 |
|  | *Scarus sp3* | Hutchin’s Parrotfish | 18 | 5.00 | 3.59 | 0.07 ±0.04 | 0.07 ±0.03 |
| Lethrinidae | *Gymnocranius grandoculis* | Robinson’s sea bream | 9 | 5.00 | 2.56 | 0.05 ±0.03 | 0.03 ±0.01 |
|  | *Lethrinus atkinsoni* | Yellow-tailed emperor | 57 |  | 3.59 |  | 0.29 ±0.13 |
|  | *Lethrinus genivittatus* | Longspine emperor | 195 | 6.67 | 14.87 | 0.10 ±0.06 | 0.97 ±0.25 |
|  | *Lethrinus laticaudis* | Grass emperor | 50 | 6.67 | 9.74 | 0.12 ±0.06 | 0.22 ±0.08 |
|  | *Lethrinus lentjan* | Pink ear emperor | 9 |  | 0.51 |  | 0.05 ±0.05 |
|  | *Lethrinus microdon* | Smalltooth emperor | 1 |  | 0.51 |  | 0.01 ±0.01 |
|  | *Lethrinus miniatus* | Trumpet emperor | 90 |  | 5.13 |  | 0.46 ±0.19 |
|  | *Lethrinus nebulosus* | Spangled emperor | 75 | 21.67 | 10.77 | 0.62 ±0.22 | 0.19 ±0.05 |
|  | *Lethrinus punctulatus* | Bluespotted emperor | 241 | 15.00 | 8.72 | 1.83 ±0.94 | 0.67 ±0.30 |
|  | *Lethrinus ravus* | Drab emperor | 25 | 3.33 | 5.64 | 0.05 ±0.04 | 0.11 ±0.04 |
|  | *Lethrinus rubrioperculatus* | Spotcheek emperor | 20 |  | 2.56 |  | 0.10 ±0.06 |
|  | *Lethrinus spp* | Emperor | 7 |  | 1.54 |  | 0.04 ±0.03 |
|  | *Lethrinus variegatus* | Variegated emperor | 26 |  | 4.62 |  | 0.13 ±0.05 |
|  | *Monotaxis grandoculis* | Big-eye bream | 1 |  | 0.51 |  | 0.01 ±0.01 |
| Lutjanidae | *Lutjanus argentimaculatus* | Mangrove jack | 1 |  | 0.51 |  | 0.01 ±0.01 |
|  | *Lutjanus carponotatus* | Spanish flag snapper | 5 | 1.67 | 1.54 | 0.02 ±0.02 | 0.02 ±0.01 |
|  | *Lutjanus erythropterus* | Crimson snapper | 43 | 13.33 | 2.05 | 0.63 ±0.44 | 0.03 ±0.01 |
|  | *Lutjanus lemniscatus* | Darktail snapper | 8 | 1.67 | 2.05 | 0.02 ±0.02 | 0.04 ±0.02 |
|  | *Lutjanus lutjanus* | Big eye snapper | 11 |  | 0.51 |  | 0.06 ±0.06 |
|  | *Lutjanus malabaricus* | Saddletail snapper | 183 | 28.33 | 3.08 | 2.92 ±0.90 | 0.04 ±0.02 |
|  | *Lutjanus quinquelineatus* | Five-lined snapper | 1 | 1.67 |  | 0.02 ±0.02 |  |
|  | *Lutjanus russellii* | Moses’ snapper | 13 | 15.00 |  | 0.22 ±0.07 |  |
|  | *Lutjanus sebae* | Red Emperor | 42 | 25.00 | 5.64 | 0.42 ±0.11 | 0.09 ±0.03 |
|  | *Lutjanus vitta* | Brownstripe snapper | 216 | 16.67 | 2.56 | 3.43 ±1.91 | 0.05 ±0.03 |
|  | *Pristipomoides multidens* | Goldband snapper | 206 | 55.00 | 23.59 | 1.55 ±0.25 | 0.58 ±0.10 |
|  | *Symphorus nematophorus* | Chinamanfish | 11 | 3.33 | 4.10 | 0.03 ±0.02 | 0.05 ±0.02 |
| Malacanthidae | *Malacanthus brevirostris* | Banded blanquillo | 3 |  | 0.51 |  | 0.02 ±0.02 |
| Microdesmidae | *Ptereleotris monoptera* | Lyretail dartfish | 38 |  | 1.54 |  | 0.19 ±0.12 |
| Mullidae | *Mulloidichthys flavolineatus* | Yellowstripe goatfish | 2 |  | 0.51 |  | 0.01 ±0.01 |
|  | *Parupeneus barberinoides* | Bicolor goatfish | 113 | 6.67 | 10.26 | 0.65 ±0.50 | 0.38 ±0.09 |
|  | *Parupeneus chrysopleuron* | Rosy goatfish | 21 | 6.67 | 2.05 | 0.18 ±0.14 | 0.05 ±0.03 |
|  | *Parupeneus cyclostomus* | Goldsaddle goatfish | 3 |  | 1.54 |  | 0.02 ±0.01 |
|  | *Parupeneus heptacanthus* | Cinnabar goatfish | 92 | 25.00 | 13.33 | 0.72 ±0.24 | 0.25 ±0.06 |
|  | *Parupeneus indicus* | Yellowspot goatfish | 45 | 23.33 | 8.21 | 0.35 ±0.09 | 0.12 ±0.04 |
|  | *Parupeneus multifasciatus* | Banded goatfish | 1 |  | 0.51 |  | 0.01 ±0.01 |
|  | *Parupeneus pleurostigma* | Sidespot goatfish | 18 | 1.67 | 5.64 | 0.02 ±0.02 | 0.09 ±0.03 |
|  | *Parupeneus spilurus* | Blackspot goatfish | 49 | 10.00 | 5.13 | 0.35 ±0.19 | 0.14 ±0.06 |
|  | *Upeneus tragula* | Bartail goatfish | 110 | 8.33 | 9.23 | 0.08 ±0.04 | 0.54 ±0.29 |
| Nemipteridae | *Nemipterus spp* | Threadfin bream | 852 | 55.00 | 64.62 | 1.73 ±0.31 | 3.84 ±0.39 |
|  | *Pentapodus emeryii* | Double whiptail | 97 | 10.00 | 11.79 | 0.17 ±0.08 | 0.45 ±0.11 |
|  | *Pentapodus porosus* | Northwest whiptail | 1009 | 26.67 | 37.44 | 2.62 ±0.71 | 4.37 ±0.55 |
|  | *Pentapodus vitta* | Black striped butterfish | 37 | 1.67 | 7.18 | 0.12 ±0.12 | 0.15 ±0.05 |
|  | *Scaevius milii* | Coral monocle bream | 3 |  | 0.51 |  | 0.02 ±0.02 |
|  | *Scolopsis monogramma* | Rainbow monocle bream | 72 | 20.00 | 13.33 | 0.47 ±0.20 | 0.23 ±0.05 |
| Pinguipedidae | *Parapercis clathrata* | Spothead grubfish | 14 | 5.00 | 4.62 | 0.05 ±0.03 | 0.06 ±0.02 |
|  | *Parapercis nebulosa* | Pinkbanded grubfish | 88 | 5.00 | 15.90 | 0.17 ±0.10 | 0.40 ±0.08 |
| Pomacanthidae | *Apolemichthys trimaculatus* | Threespot angelfish | 1 |  | 0.51 |  | 0.01 ±0.01 |
|  | *Chaetodontoplus duboulayi* | Scribbled angelfish | 28 | 3.33 | 9.23 | 0.03 ±0.02 | 0.13 ±0.03 |
|  | *Chaetodontoplus personifer* | Yellowtail angelfish | 21 | 5.00 | 4.62 | 0.07 ±0.04 | 0.09 ±0.03 |
|  | *Pomacanthus imperator* | Emperor angelfish | 4 | 1.67 | 1.54 | 0.02 ±0.02 | 0.02 ±0.01 |
|  | *Pomacanthus semicirculatus* | Semicircle angelfish | 15 | 3.33 | 4.62 | 0.03 ±0.02 | 0.07 ±0.02 |
|  | *Pomacanthus sexstriatus* | Sixbar angelfish | 14 | 1.67 | 3.59 | 0.03 ±0.03 | 0.06 ±0.02 |
| Pomacentridae | *Abudefduf bengalensis* | Bengal sergeant | 13 | 6.67 | 0.51 | 0.20 ±0.12 | 0.01 ±0.01 |
|  | *Amblypomacentrus breviceps* | Blackbanded damsel | 1 |  | 0.51 |  | 0.01 ±0.01 |
|  | *Amphiprion clarkii* | Clark’s anemonefish | 5 |  | 0.51 |  | 0.03 ±0.03 |
|  | *Chromis fumea* | Smokey puller | 93 | 1.67 | 4.10 | 0.02 ±0.02 | 0.47 ±0.22 |
|  | *Dascyllus reticulatus* | Headband humbug | 2 |  | 0.51 |  | 0.01 ±0.01 |
|  | *Dascyllus trimaculatus* | Threespot humbug | 3 | 1.67 |  | 0.05 ±0.05 |  |
|  | *Pomacentrus coelestis* | Neon damsel | 285 |  | 6.15 |  | 1.46 ±0.66 |
|  | *Pomacentrus milleri* | Miller’s damsel | 5 |  | 1.03 |  | 0.03 ±0.02 |
|  | *Pomacentrus nagasakiensis* | Blue-scribbled damsel | 27 | 3.33 | 1.03 | 0.10 ±0.07 | 0.11 ±0.09 |
|  | *Pomacentrus vaiuli* | Princess damsel | 12 | 1.67 | 1.54 | 0.05 ±0.05 | 0.05 ±0.03 |
|  | *Pristotis obtusirostris* | Gulf damsel | 22 | 1.67 | 2.05 | 0.02 ±0.02 | 0.11 ±0.07 |
| Rachycentridae | *Rachycentron canadum* | Cobia | 5 | 5.00 | 1.03 | 0.05 ±0.03 | 0.01 ±0.01 |
| Scombridae | *Scomberomorus spp* | Mackerel | 123 | 18.33 | 36.41 | 0.20 ±0.06 | 0.57 ±0.07 |
| Siganidae | *Siganus argenteus* | Forktail rabbitfish | 2 |  | 0.51 |  | 0.01 ±0.01 |
|  | *Siganus doliatus* | Barred rabbitfish | 7 |  | 2.05 |  | 0.04 ±0.02 |
|  | *Siganus fuscescens* | Black rabbitfish | 156 | 11.67 | 7.69 | 0.77 ±0.56 | 0.56 ±0.21 |
| Sillaginidae | *Sillago spp* | Whiting | 9 | 1.67 | 1.54 | 0.03 ±0.03 | 0.04 ±0.02 |
| Sparidae | *Argyrops spinifer* | Frypan snapper | 151 | 53.33 | 24.62 | 1.02 ±0.15 | 0.46 ±0.08 |
| Sphyraenidae | *Sphyraena barracuda* | Great barracuda | 3 | 1.67 | 1.03 | 0.02 ±0.02 | 0.01 ±0.01 |
|  | *Sphyraena jello* | Pickhandle barracuda | 2 |  | 1.03 |  | 0.01 ±0.01 |
|  | *Sphyraena obtusata* | Yellowtail barracuda | 22 | 1.67 |  | 0.37 ±0.37 |  |
|  | *Sphyraena qenie* | Blackfin barracuda | 3 |  | 0.51 |  | 0.02 ±0.02 |
| Terapontidae | *Terapon jarbua* | Crescent grunter | 192 |  | 13.85 |  | 0.98 ±0.28 |
| Tripterygiidae | *Tripterygiidae spp* | Triplefin blennies | 3 |  | 0.51 |  | 0.02 ±0.02 |
| ***Pleuroneciformes*** |  |  |  |  |  |  |  |
| Bothidae | *Bothidae spp* | Lefteye flounders | 6 | 3.33 | 2.05 | 0.03 ±0.02 | 0.02 ±0.01 |
| ***Scorpaeniformes*** |  |  |  |  |  |  |  |
| Platycephalidae | *Platycephalus spp* | Flatheads | 2 |  | 0.51 |  | 0.01 ±0.01 |
| Pteroidae | *Pterois volitans* | Red lionfish | 1 |  | 0.51 |  | 0.01 ±0.01 |
| ***Siluriformes*** |  |  |  |  |  |  |  |
| Ariidae | *Netuma thalassina* | Giant sea catfish | 43 | 13.33 | 11.79 | 0.18 ±0.08 | 0.16 ±0.04 |
| Plotosidae | *Paraplotosus butleri* | Sailfin catfish | 1 | 1.67 |  | 0.02 ±0.02 |  |
| ***Sygnathiformes*** |  |  |  |  |  |  |  |
| Fistulariidae | *Fistularia commersonii* | Smooth flutemouth | 3 |  | 1.54 |  | 0.02 ±0.01 |
| ***Tetradontiformes*** |  |  |  |  |  |  |  |
| Diodontidae | *Diodon hystrix* | Spotted porcupinefish | 1 |  | 0.51 |  | 0.01 ±0.01 |
| Balistidae | *Pseudobalistes fuscus* | Yellowspotted triggerfish | 11 | 6.67 | 3.08 | 0.08 ±0.04 | 0.03 ±0.01 |
|  | *Sufflamen chrysopterum* | Halfmoon triggerfish | 9 |  | 2.56 |  | 0.05 ±0.02 |
|  | *Sufflamen fraenatum* | Masked triggerfish | 71 | 21.67 | 13.33 | 0.45 ±0.13 | 0.23 ±0.04 |
|  | *Abalistes stellatus* | Starry triggerfish | 80 | 23.33 | 24.62 | 0.28 ±0.08 | 0.32 ±0.05 |
| Monacanthidae | *Anacanthus barbatus* | Bearded leatherjacket | 2 |  | 0.51 |  | 0.01 ±0.01 |
|  | *Monacanthus chinensis* | Fanbelly leatherjacket | 4 |  | 2.05 |  | 0.02 ±0.01 |
|  | *Paramonacanthus choirocephalus* | Pigface leatherjacket | 52 | 3.33 | 13.33 | 0.05 ±0.04 | 0.25 ±0.06 |
| Ostraciidae | *Ostracion cubicus* | Yellow boxfish | 1 |  | 0.51 |  | 0.01 ±0.01 |
| Tetradontidae | *Canthigaster valentini* | Blacksaddle toby | 7 | 5.00 | 2.05 | 0.05 ±0.03 | 0.02 ±0.01 |
|  | *Feroxodon multistriatus* | Many-stripped puffer | 2 |  | 1.03 |  | 0.01 ±0.01 |
|  | *Lagocephalus lunaris* | Lunartail puffer | 86 | 10.00 | 28.72 | 0.13 ±0.06 | 0.40 ±0.05 |
|  | *Lagocephalus sceleratus* | Northwest Blowie | 51 | 5.00 | 14.36 | 0.05 ±0.03 | 0.25 ±0.05 |
|  | *Torquigener pallimaculatus* | Rusty-spotted toadfish | 3 |  | 1.03 |  | 0.02 ±0.01 |
