# Supplementary material for: The influence of depth and a subsea pipeline on fish assemblages and commercially fished species
Source: PLoS One. 2018 Nov 26;13(11):e0207703. doi: 10.1371/journal.pone.0207703 (PMC6257935; doi:10.1371/journal.pone.0207703)
Supplement: S2 Table — Species that appear as critically endangered (CE), endangered (EN), vulnerable (VU), or near threatened (NT) on the IUCN Red List are identified. Species listed as “sp10” were not identifiable to species level but are included as they were different species to all others listed. (DOCX) [file pone.0207703.s002.docx]

S-Table 2: The abundance and commonality (% deployments) of all cartilaginous fish species (alphabetical by Order then family and then genus) observed on the Griffin pipeline and those in the surrounding natural environment (Off pipeline). Species that appear as critically endangered (*CE*), endangered (*EN*), vulnerable (*VU*), or near threatened (*NT)* on the IUCN Red List are identified. Species listed as “sp10” were not identifiable to species level but are included as they were different species to all others listed.

| ***Order***/Family | Species | Common Name | Total Abundance | Commonality (%) | | Mean Abundance ±SE | |
| --- | --- | --- | --- | --- | --- | --- | --- |
|  |  |  |  | Pipeline | Off-pipeline | Pipeline | Off-pipeline |
| ***Carcharhiniformes*** |  |  |  |  |  |  |  |
| Carcharhinidae | *Carcharhinus amblyrhynchos ^NT^* | Grey reef shark | 14 | 1.67 | 4.10 | 0.02 ±0.02 | 0.07 ±0.03 |
|  | *Carcharhinus amboinensis* | Pigeye shark | 1 |  | 0.51 |  | 0.01 ±0.01 |
|  | *Carcharhinus coatesi ^NT^* | White cheek shark | 5 | 1.67 | 2.05 | 0.02 ±0.02 | 0.02 ±0.01 |
|  | *Carcharhinus limbatus ^NT^* | Blacktip shark | 3 | 3.33 | 0.51 | 0.03 ±0.02 | 0.01 ±0.01 |
|  | *Carcharhinus macloti ^NT^* | Hardnose shark | 2 |  | 1.03 |  | 0.01 ±0.01 |
|  | *Carcharhinus melanopterus ^NT^* | Blacktip reef shark | 2 |  | 1.03 |  | 0.01 ±0.01 |
|  | *Carcharhinus plumbeus ^VU^* | Sandbar shark | 19 | 13.33 | 5.64 | 0.13 ±0.04 | 0.06 ±0.02 |
|  | *Carcharhinus sorrah ^NT^* | Spot-tail shark | 21 | 6.67 | 7.69 | 0.07 ±0.03 | 0.09 ±0.02 |
|  | *Carcharhinus tilstoni* | Australian blacktip shark | 11 | 3.33 | 4.10 | 0.03 ±0.02 | 0.05 ±0.02 |
|  | *Carcharhinus sp10* | Requiem shark | 6 |  | 3.08 |  | 0.03 ±0.01 |
|  | *Galeocerdo cuvier ^NT^* | Tiger shark | 4 | 1.67 | 1.54 | 0.02 ±0.13 | 0.02 ±0.01 |
|  | *Loxodon macrorhinus* | Sliteye shark | 35 | 10.00 | 12.31 | 0.15 ±0.42 | 0.10 ±0.30 |
|  | *Negaprion acutidens^VU^* | Sicklefin lemon shark | 2 |  | 1.03 |  | 0.01 ±0.01 |
|  | *Triaenodon obesus ^NT^* | Whitetip reef shark | 1 |  | 0.51 |  | 0.01 ±0.01 |
| Hemigaleidae | *Hemipristis elongata ^VU^* | Fossil shark | 2 |  | 1.03 |  | 0.01 ±0.01 |
| Sphyrinidae | *Sphyrna lewini ^EN^* | Scalloped hammerhead | 3 | 3.33 | 0.51 | 0.03 ±0.02 | 0.01 ±0.01 |
|  | *Sphyrna mokarran ^EN^* | Great hammerhead | 7 | 3.33 | 2.56 | 0.03 ±0.02 | 0.03 ±0.01 |
| ***Pristiformes*** |  |  |  |  |  |  |  |
| Pristidae | *Pristis zijsron ^CE^* | Longcomb sawfish | 1 | 1.67 |  | 0.02 ±0.02 |  |
| ***Orectolobiformes*** |  |  |  |  |  |  |  |
| Ginglymostomatidae | *Nebrius ferrugineus ^VU^* | Tawny nurse shark | 4 |  | 2.05 |  | 0.02 ±0.01 |
| Hemiscylliidae | *Chiloscyllium punctatum ^NT^* | Grey carpetshark | 1 |  | 0.51 |  | 0.01 ±0.01 |
| Stegostomatidae | *Stegostoma fasciatum ^EN^* | Zebra shark | 8 | 1.67 | 3.59 | 0.02 ±0.02 | 0.04 ±0.01 |
| ***Rajiformes*** |  |  |  |  |  |  |  |
| Rhinobatidae | *Glaucostegus typus ^VU^* | Giant shovelnose ray | 2 | 1.67 | 0.51 | 0.02 ±0.02 | 0.01 ±0.01 |
|  | *Rhynchobatus australiae ^VU^* | Whitespotted guitarfish | 10 | 1.67 | 4.62 | 0.02 ±0.02 | 0.05 ±0.02 |
| ***Myliobatiformes*** |  |  |  |  |  |  |  |
| Dasyatidae | *Himantura uarnak ^VU^* | Honeycomb stingray | 1 |  | 0.51 |  | 0.01 ±0.01 |
|  | *Neotrygon australiae* | Bluespotted maskray | 1 | 1.67 |  | 0.02 ±0.02 |  |
|  | *Paleobatis fai ^VU^* | Pink whipray | 1 |  | 0.51 |  | 0.01 ±0.01 |
|  | *Paleobatis jenkinsii ^VU^* | Jenkins’ whipray | 1 |  | 0.51 |  | 0.01 ±0.01 |
|  | *Pastinachus ater* | Cowtail stingray | 1 |  | 0.51 |  | 0.01 ±0.01 |
|  | *Taeniurops meyeni ^VU^* | Round ribbontail ray | 2 |  | 1.03 |  | 0.01 ±0.01 |
