# Supplementary material for: The influence of depth and a subsea pipeline on fish assemblages and commercially fished species
Source: PLoS One. 2018 Nov 26;13(11):e0207703. doi: 10.1371/journal.pone.0207703 (PMC6257935; doi:10.1371/journal.pone.0207703)
Supplement: S3 Table — The results of subsequent depth range analyses results are also presented. Abundance results are in blue and biomass in orange. (DOCX) [file pone.0207703.s003.docx]

S-Table 3: Results of PERMANOVA analyses on the fish assemblage relative abundance and biomass (multivariate analyses; fourth-root transformed data, Bray Curtis dissimilarities) and on the total abundance and biomass of commercial species (univariate analyses; fourth-root transformed, Euclidean Distance dissimiliarities). The results of subsequent depth range analyses results are also presented. Abundance results are in blue and biomass in orange.

| **Entire Fish Assemblage (multivariate)** | | | |  |  |  |  |  |  |  |  |  |  |  |  |
| --- | --- | --- | --- | --- | --- | --- | --- | --- | --- | --- | --- | --- | --- | --- | --- |
|  | **Relative abundance** | | | | | **Biomass** | | | | |  |  |  |  |  |
| **Source** | **df** | **MS** | **Pseudo-F** | **P(perm)** | **Unique perms** | **df** | **MS** | **Pseudo-F** | **P(perm)** | **Unique perms** |  |  |  |  |  |
| **IvC** | 1 | 28099 | 4 | **0.014** | 9946 | 1 | 7136.6 | 2.145 | 0.072 | 9944 |  |  |  |  |  |
| **Lo(IvC)** | 1 | 7471 | 0.751 | 0.587 | 9925 | 1 | 3441.5 | 0.779 | 0.626 | 9919 |  |  |  |  |  |
| **Si(Lo(IvC))** | 51 | 9782.6 | 5.353 | **<0.001** | 9625 | 51 | 4372.9 | 2.753 | **<0.001** | 9548 |  |  |  |  |  |
| **Res** | 201 | 1827.5 |  |  |  | 194 | 1588.3 |  |  |  |  |  |  |  |  |
| **Total** | 254 |  |  |  |  | 247 |  |  |  |  |  |  |  |  |  |
|  |  |  |  |  |  |  |  |  |  |  |  |  |  |  |  |
|  | **Relative abundance <40 m** | | | | | **Relative abundance 40-80 m** | | | | | **Relative abundance >80 m** | | | | |
| **Source** | **df** | **MS** | **Pseudo-F** | **P(perm)** | **Unique perms** | **df** | **MS** | **Pseudo-F** | **P(perm)** | **Unique perms** | **df** | **MS** | **Pseudo-F** | **P(perm)** | **Unique perms** |
| **IvC** | 1 | 7768 | 0.966 | 0.453 | 9948 | 1 | 9818.9 | 2.728 | 0.067 | 6892 | 1 | 20778 | 4.078 | **0.048** | 9949 |
| **Lo(IvC)** | 1 | 9611.7 | 1.514 | 0.144 | 9917 | 1 | 3812 | 0.798 | 0.624 | 6915 | 1 | 5797.8 | 1.264 | 0.26 | 9945 |
| **Si(Lo(IvC))** | 21 | 6355.3 | 2.673 | **<0.01** | 9735 | 7 | 4657 | 2.789 | **<0.001** | 9844 | 18 | 4259.4 | 3.745 | **<0.001** | 9820 |
| **Res** | 95 | 2377.5 |  |  |  | 38 | 1669.5 |  |  |  | 67 | 1137.5 |  |  |  |
| **Total** | 118 |  |  |  |  | 47 |  |  |  |  | 87 |  |  |  |  |
|  |  |  |  |  |  |  |  |  |  |  |  |  |  |  |  |
| **Totals for Commercial Species (univariate)** | | | |  |  |  |  |  |  |  |  |  |  |  |  |
|  | **Total abundance** | | | | | **Total Biomass** | | | | |  |  |  |  |  |
| **Source** | **df** | **MS** | **Pseudo-F** | **P(perm)** | **Unique perms** | **df** | **MS** | **Pseudo-F** | **P(perm)** | **Unique perms** |  |  |  |  |  |
| **IvC** | 1 | 26.522 | 69.871 | **<0.001** | 9869 | 1 | 8.679 | 35.741 | **<0.001** | 9864 |  |  |  |  |  |
| **Lo(IvC)** | 1 | 0.18 | 0.171 | 0.671 | 9821 | 1 | 7.60E-02 | 0.116 | 0.731 | 9849 |  |  |  |  |  |
| **Si(Lo(IvC))** | 51 | 1.072 | 2.843 | **<0.001** | 9879 | 51 | 0.702 | 1.807 | **<0.01** | 9862 |  |  |  |  |  |
| **Res** | 201 | 0.377 |  |  |  | 194 | 0.388 |  |  |  |  |  |  |  |  |
| **Total** | 254 |  |  |  |  | 247 |  |  |  |  |  |  |  |  |  |
|  |  |  |  |  |  |  |  |  |  |  |  |  |  |  |  |
|  | **Total abundance <40 m** | | | | | **Total abundance 40-80 m** | | | | | **Total abundance >80 m** | | | | |
| **Source** | **df** | **MS** | **Pseudo-F** | **P(perm)** | **Unique perms** | **df** | **MS** | **Pseudo-F** | **P(perm)** | **Unique perms** | **df** | **MS** | **Pseudo-F** | **P(perm)** | **Unique perms** |
| **IvC** | 1 | 4.561 | 5.418 | **0.051** | 9867 | 1 | 12.659 | 89.683 | **0.002** | 6755 | 1 | 7.320 | 60.962 | 0.089 | 9972 |
| **Lo(IvC)** | 1 | 0.701 | 0.72 | 0.399 | 9841 | 1 | 0.02 | 0.033 | 0.867 | 5373 | 1 | 0.081 | 0.106 | 0.756 | 9846 |
| **Si(Lo(IvC))** | 21 | 0.974 | 1.898 | **0.02** | 9917 | 7 | 1.008 | 2.766 | **0.021** | 9938 | 18 | 0.858 | 4.364 | **<0.001** | 9930 |
| **Res** | 95 | 0.513 |  |  |  | 38 | 0.364 |  |  |  | 67 | 0.197 |  |  |  |
| **Total** | 118 |  |  |  |  | 47 |  |  |  |  | 87 |  |  |  |  |
|  |  |  |  |  |  |  |  |  |  |  |  |  |  |  |  |
|  | **Total biomass <40 m** | | | | | **Total biomass 40-80 m** | | | | | **Total biomass >80 m** | | | | |
| **Source** | **df** | **MS** | **Pseudo-F** | **P(perm)** | **Unique perms** | **df** | **MS** | **Pseudo-F** | **P(perm)** | **Unique perms** | **df** | **MS** | **Pseudo-F** | **P(perm)** | **Unique perms** |
| **IvC** | 1 | 1.589 | 3.972 | 0.096 | 9843 | 1 | 4.179 | 11.080 | 0.092 | 6751 | 1 | 1.856 | 11.217 | 0.197 | 9968 |
| **Lo(IvC)** | 1 | 0.060 | 0.081 | 0.775 | 9830 | 1 | 0.300 | 0.283 | 0.599 | 5358 | 1 | 0.069 | 0.208 | 0.659 | 9842 |
| **Si(Lo(IvC))** | 21 | 0.752 | 1.823 | **0.028** | 9929 | 7 | 1.078 | 2.553 | **0.031** | 9946 | 18 | 0.458 | 1.359 | 0.183 | 9925 |
| **Res** | 90 | 0.412 |  |  |  | 38 | 0.422 |  |  |  | 65 | 0.337 |  |  |  |
| **Total** | 113 |  |  |  |  | 47 |  |  |  |  | 85 |  |  |  |  |
|  |  |  |  |  |  |  |  |  |  |  |  |  |  |  |  |
