# Supplementary material for: The influence of depth and a subsea pipeline on fish assemblages and commercially fished species
Source: PLoS One. 2018 Nov 26;13(11):e0207703. doi: 10.1371/journal.pone.0207703 (PMC6257935; doi:10.1371/journal.pone.0207703)
Supplement: S4 Table — Key species are divided into <40m 40-80m and >80 m depth categories depending on the direction of the vectors in the CAP. eDF, estimated degrees of freedom; AIC, Akaike Information Criterion; wAIC, Weighted Akaike Information Criterion. (DOCX) [file pone.0207703.s004.docx]

S-Table 4. Best generalised additive models (GAMs) for predicting total abundance, species richness, and the abundance of key species identified in Figure 2. Key species are divided into <40m 40-80m and >80 m depth categories depending on the direction of the vectors in the CAP. eDF, estimated degrees of freedom; AIC, Akaike Information Criterion; wAIC, Weighted Akaike Information Criterion.

|  | **Dependent variable** | **eDF** | **AIC** | **wAIC** | **R^2^** | **Best Model** |
| --- | --- | --- | --- | --- | --- | --- |
|  | Total Abundance | 5.30 | 2413.79 | 0.39 | 0.49 | Depth + Mean relief |
|  | Species Richness | 4.45 | 1435.57 | 0.92 | 0.50 | Depth + Mean relief |
| <40 m | *Pentapodus porosus* | 3.97 | 771.28 | 0.37 | 0.68 | Depth + Benthic biota |
|  | *Selaroides leptolepis* | 4.23 | 792.50 | 0.50 | 0.59 | Depth + Benthic biota |
|  | *Scomberomorus* spp. | 5.35 | 391.69 | 0.60 | 0.10 | Depth + Unconsolidated |
| 40-80 m | *Carangoides caeruleopinnatus* | 7.95 | 488.01 | 0.65 | 0.37 | Depth + Mean relief |
|  | *Carangoides chrysophrys* | 2.00 | 443.46 | 0.51 | 0.03 | Mean relief |
|  | *Nemipterus* spp. | 6.49 | 1059.17 | 0.85 | 0.39 | Depth + Mean relief |
| >80 m | *Argyrops spinifer* | 6.43 | 505.31 | 0.48 | 0.15 | Minimum distance + Unconsolidated |
|  | *Decapterus s*p1 | 3.88 | 559.44 | 0.50 | 0.54 | Depth |
|  | *Pristipomoides multidens* | 3.73 | 472.97 | 0.57 | 0.63 | Depth |
