# Supplementary material for: The influence of depth and a subsea pipeline on fish assemblages and commercially fished species
Source: PLoS One. 2018 Nov 26;13(11):e0207703. doi: 10.1371/journal.pone.0207703 (PMC6257935; doi:10.1371/journal.pone.0207703)
Supplement: S5 Table — (DOCX) [file pone.0207703.s005.docx]

S-Table 5: Summary of the commercial catches and the relative contribution (% composition) of each of the major or iconic species taken within the Pilbara and Kimberley sectors of the North Coast Bioregion in 2015 (Fletcher et al. 2017)

|  |  | Pilbara Catch | | Kimberly (NDSF) catch | | Total catch (tonnes) |
| --- | --- | --- | --- | --- | --- | --- |
| Common name | Species | tonnes | % total | tonnes | % total |  |
| Frypan snapper | *Argyrops spinifer* | 29.3 | >99 | <0.1 | <1 | 29.3 |
| Rankin cod | *Epinephelus multinotatus* | 84 | 62 | 51.5 | 38 | 135.5 |
| Robinson’s seabream | *Gymnocranius grandoculis* | 24.8 | 88 | 3.5 | 12 | 28.3 |
| Grass emperor | *Lethrinus laticaudis* | 0.3 | 13 | 2.1 | 88 | 2.4 |
| Spangled emperor | *Lethrinus nebulosus* | 44.4 | 72 | 16.9 | 28 | 61.3 |
| Longnose emperor | *Lethrinus olivaceus* | 7 | 66 | 3.6 | 34 | 10.6 |
| Bluespotted emperor | *Lethrinus punctulatus* | 238.8 | 84 | 45.1 | 16 | 283.9 |
| Crimson snapper | *Lutjanus erythropterus* | 158.6 | 80 | 38.7 | 20 | 197.3 |
| Saddletail snapper | *Lutjanus malabaricus* | 74.1 | 43 | 99.4 | 57 | 173.5 |
| Moses snapper | *Lutjanus russellii* | 29.5 | 69 | 13.2 | 31 | 42.7 |
| Red emperor | *Lutjanus sebae* | 116.4 | 47 | 131.7 | 53 | 248.1 |
| Brownstripe snapper | *Lutjanus vitta* | 112.2 | 86 | 18.3 | 14 | 130.5 |
| Rosy threadfin bream | *Nemipterus furcosus* | 86.5 | >99 | 0.2 | <1 | 86.7 |
| Barcheek coral trout | *Plectropomus* spp*.* | 15.5 | 81 | 3.7 | 19 | 19.2 |
| Goldband snapper | *Pristipomoides multidens* | 208.9 | 31 | 457.2 | 69 | 666.1 |
| **Total all demersal scalefish** | | **1779.3** | **63** | **1046.4** | **37** | **2825.7** |
