# Supplementary figures and images for: The influence of depth and a subsea pipeline on fish assemblages and commercially fished species
Source: PLoS One. 2018 Nov 26;13(11):e0207703. doi: 10.1371/journal.pone.0207703 (PMC6257935; doi:10.1371/journal.pone.0207703)

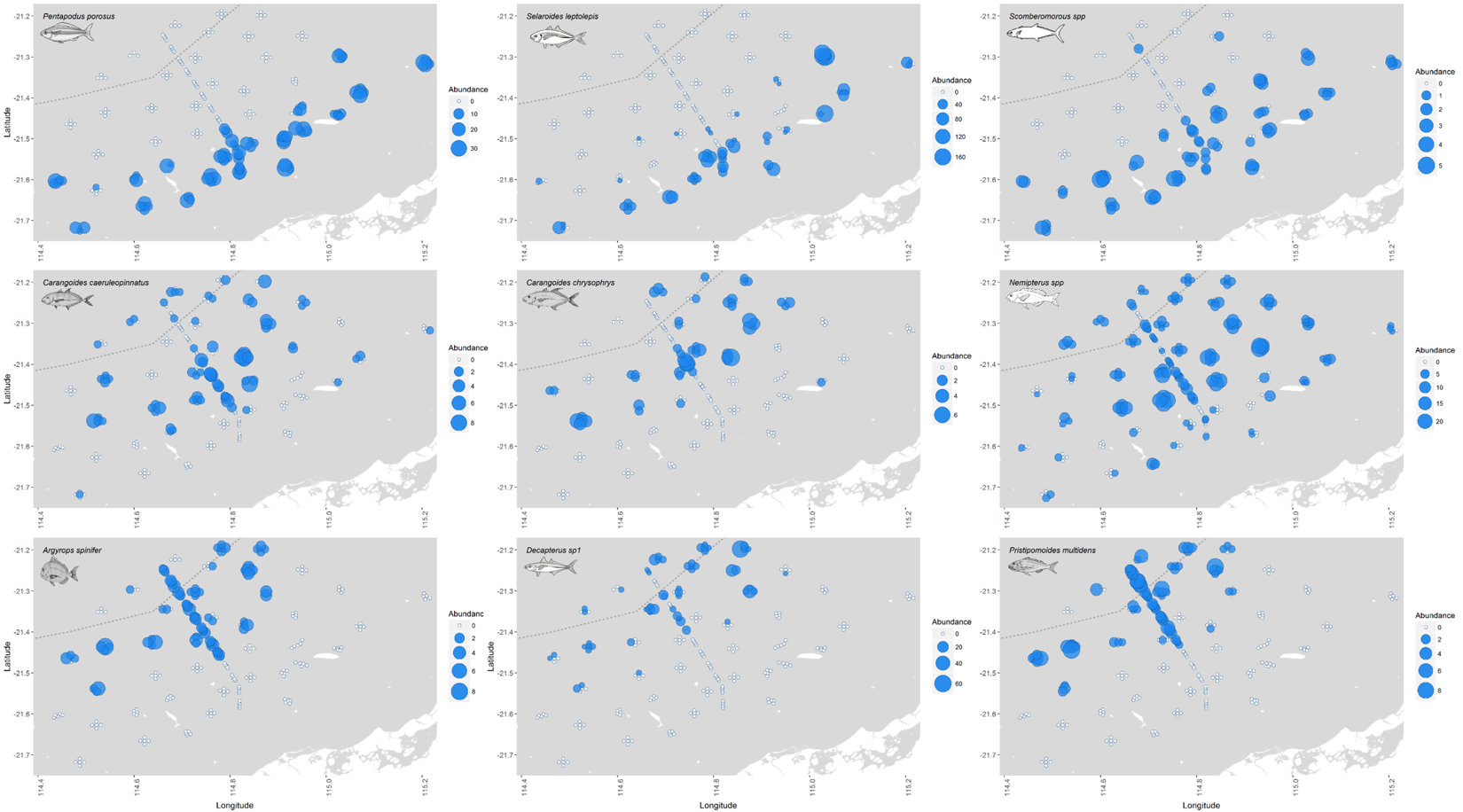

Supplement: S1 Fig — MaxN bubble sizes reflect exact abundance, and therefore bubble size may be larger or smaller than those in the legend’s categories. (TIF) [file pone.0207703.s006.tif]

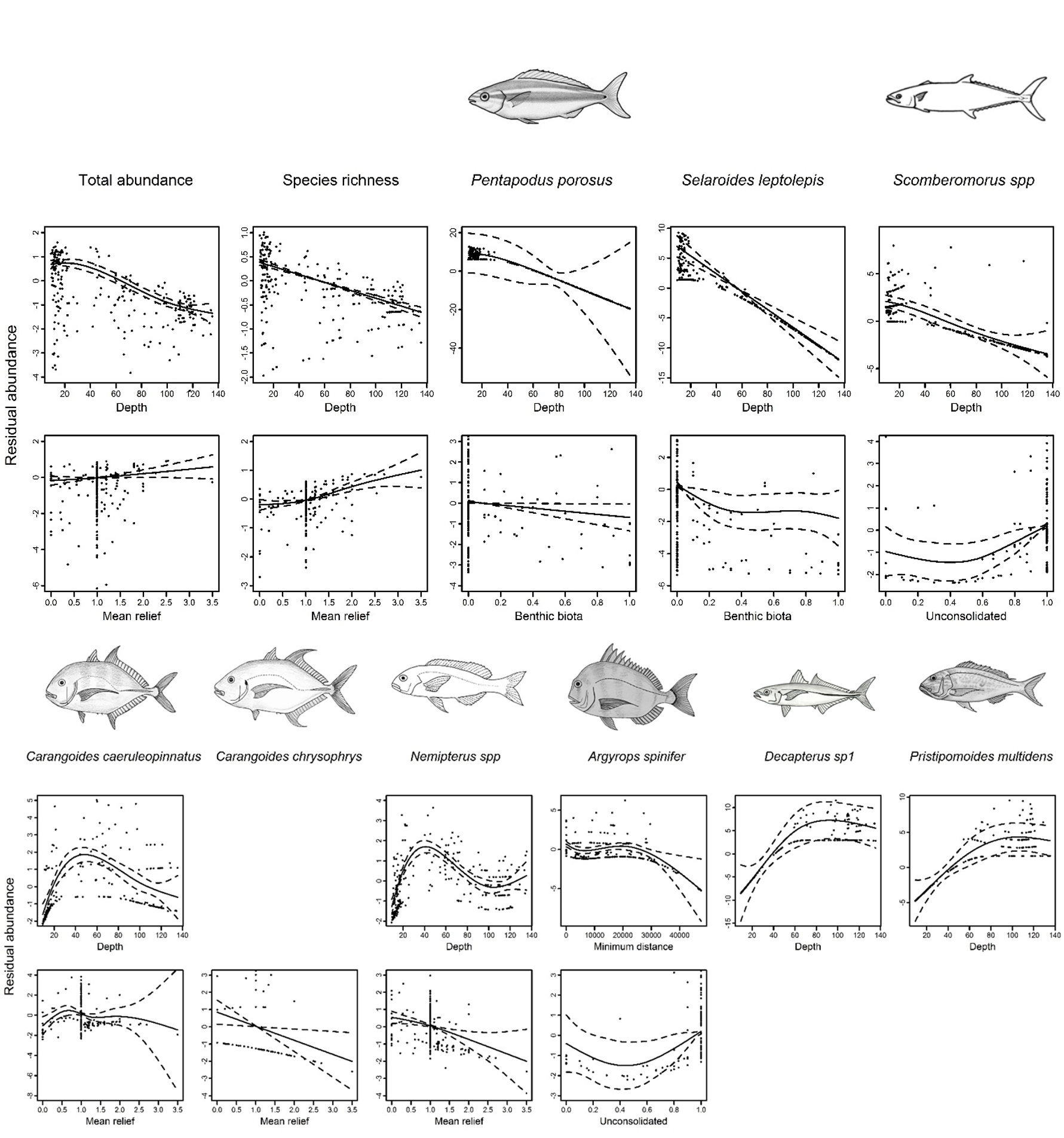

Supplement: S2 Fig — The solid black line represents the estimated smoothing curve and dashed lines represents ±2 x SE of the estimate. (TIF) [file pone.0207703.s007.tif]

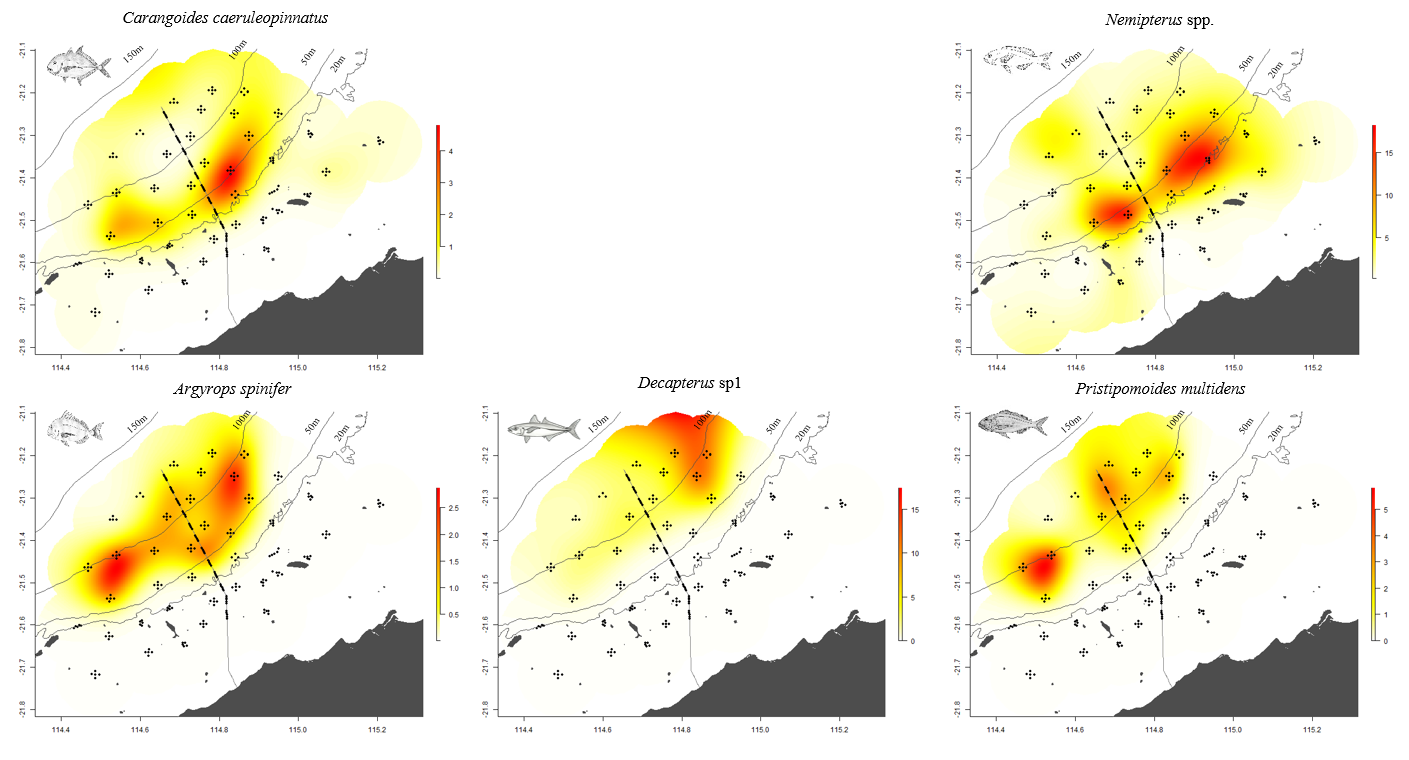

Supplement: S3 Fig — Colour ramp represents the abundance predicted by latitude and longitude alone. Heatmaps of other common and interesting species are presented in Appendix 4. (TIFF) [file pone.0207703.s008.tiff]

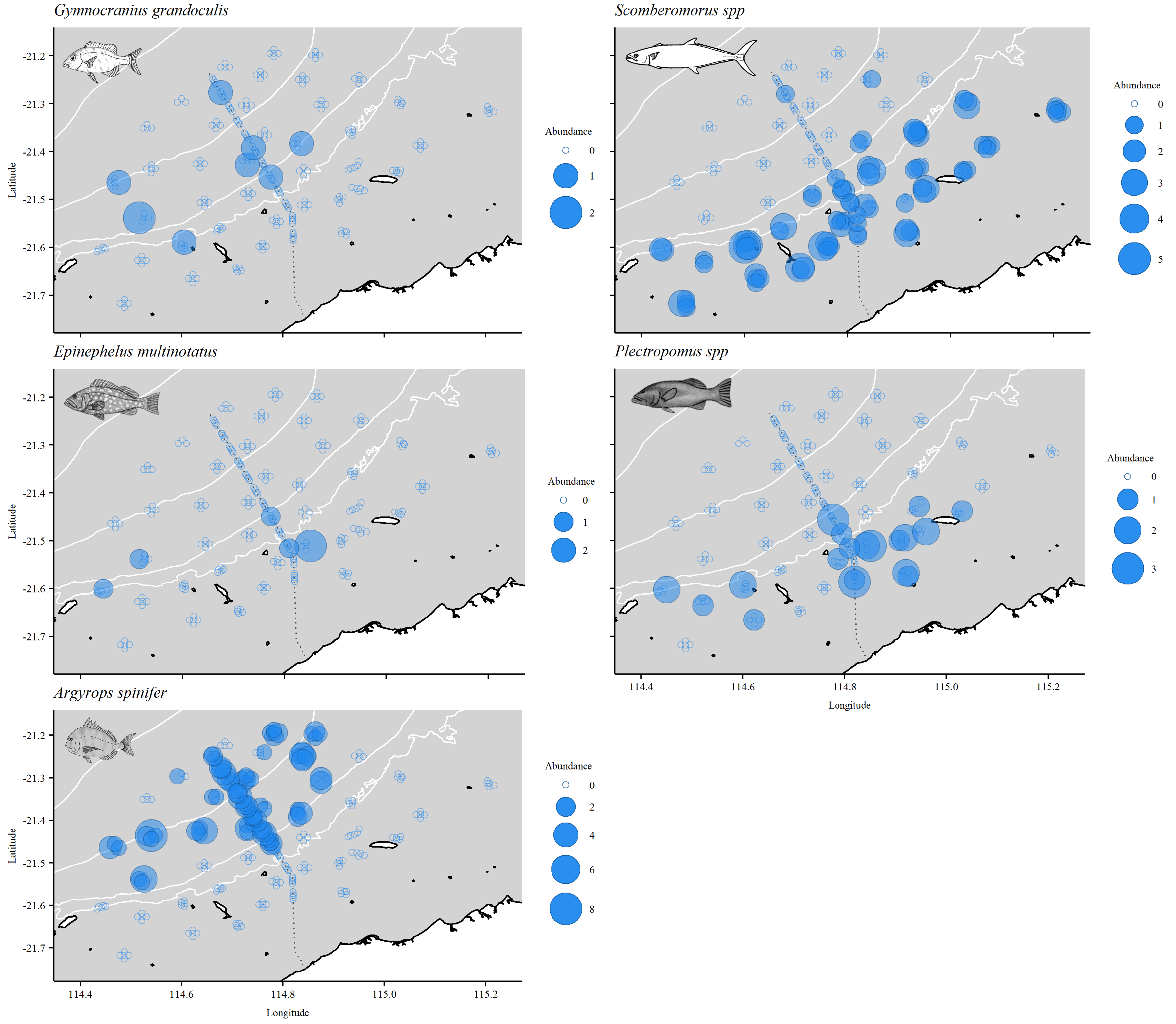

Supplement: S5 Fig — MaxN bubble sizes reflect actual abundance, and therefore bubble size may be larger or smaller than those in the legend. (TIFF) [file pone.0207703.s010.tiff]
